# Effects of exercise on NAFLD using non-targeted metabolomics in adipose tissue, plasma, urine, and stool

**DOI:** 10.1038/s41598-022-10481-9

**Published:** 2022-04-20

**Authors:** Ambrin Farizah Babu, Susanne Csader, Ville Männistö, Milla-Maria Tauriainen, Heikki Pentikäinen, Kai Savonen, Anton Klåvus, Ville Koistinen, Kati Hanhineva, Ursula Schwab

**Affiliations:** 1 Department of Public Health and Clinical Nutrition, University of Eastern Finland, 70210 Kuopio, Finland; 2 Afekta Technologies Ltd., Yliopistonranta 1L, 70211 Kuopio, Finland; 3 Department of Medicine, University of Eastern Finland and Kuopio University Hospital, Kuopio, Finland; 4 Kuopio Research Institute of Exercise Medicine, Kuopio, Finland; 5 Department of Clinical Physiology and Nuclear Medicine, Kuopio University Hospital, Kuopio, Finland; 6 Department of Life Technologies, Food Chemistry and Food Development Unit, University of Turku, 20014 Turku, Finland; 7 Department of Medicine, Endocrinology and Clinical Nutrition, Kuopio University Hospital, Kuopio, Finland

**Keywords:** Fat metabolism, Metabolomics, Randomized controlled trials

## Abstract

The mechanisms by which exercise benefits patients with non-alcoholic fatty liver disease (NAFLD), the most common liver disease worldwide, remain poorly understood. A non-targeted liquid chromatography-mass spectrometry (LC–MS)-based metabolomics analysis was used to identify metabolic changes associated with NAFLD in humans upon exercise intervention (without diet change) across four different sample types—adipose tissue (AT), plasma, urine, and stool. Altogether, 46 subjects with NAFLD participated in this randomized controlled intervention study. The intervention group (n = 21) performed high-intensity interval training (HIIT) for 12 weeks while the control group (n = 25) kept their sedentary lifestyle. The participants' clinical parameters and metabolic profiles were compared between baseline and endpoint. HIIT significantly decreased fasting plasma glucose concentration (p = 0.027) and waist circumference (p = 0.028); and increased maximum oxygen consumption rate and maximum achieved workload (p < 0.001). HIIT resulted in sample-type-specific metabolite changes, including accumulation of amino acids and their derivatives in AT and plasma, while decreasing in urine and stool. Moreover, many of the metabolite level changes especially in the AT were correlated with the clinical parameters monitored during the intervention. In addition, certain lipids increased in plasma and decreased in the stool. Glyco-conjugated bile acids decreased in AT and urine. The 12-week HIIT exercise intervention has beneficial ameliorating effects in NAFLD subjects on a whole-body level, even without dietary changes and weight loss. The metabolomics analysis applied to the four different sample matrices provided an overall view on several metabolic pathways that had tissue-type specific changes after HIIT intervention in subjects with NAFLD. The results highlight especially the role of AT in responding to the HIIT challenge, and suggest that altered amino acid metabolism in AT might play a critical role in e.g. improving fasting plasma glucose concentration.

**Trial registration** ClinicalTrials.gov (NCT03995056).

## Introduction

Approximately 25% of the population worldwide is affected by non-alcoholic fatty liver disease (NAFLD), making it the most common liver disease worldwide, and a major public health concern^1,2^. NAFLD encompasses liver conditions ranging from steatosis through steatohepatitis to liver cirrhosis^3^. NAFLD is also associated with insulin resistance and components of metabolic syndrome, including obesity, type 2 diabetes (T2D), and hyperlipidemia, thereby making NAFLD a multisystem disease^4^.

Physical exercise is a first-line therapy for patients with NAFLD. Clinical studies focusing on physical exercise interventions have shown that exercise decreases weight, waist circumference, body fat, and blood pressure in NAFLD patients^5^. Moreover, clinical parameters of NAFLD, such as intrahepatic lipid content (IHL), insulin sensitivity, and liver enzymes such as alanine aminotransaminase (ALT) and aspartate aminotransaminase (AST) have also been improved with physical exercise^6–10^. Furthermore, changes in the levels of endogenous and gut-microbiota produced metabolites including purine, tryptophan, carnitine, and steroid metabolites induced by physical exercise may be partially responsible for the exercise-related health benefits^11,12^. However, the mechanisms behind such improvements and the contribution of these metabolites in the context of NAFLD are poorly understood.

High-throughput metabolomics technology has the potential to offer a comprehensive view of the metabolic changes related to NAFLD and has indeed already proposed various biomarkers for NAFLD, including amino acids, bile acids, phosphocholines, carbohydrates, and gut microbiota-produced metabolites such as succinic acid, phenylacetic acid, and 3-(4-hydroxyphenyl) lactic acid^13–17^. Non‐targeted metabolite profiling could likewise serve as a valuable tool for assessing metabolic differences among NAFLD patients in response to exercise and may help uncover a wide spectrum of endogenous and gut microbial metabolites altered during exercise. In this study, we utilized a non-targeted metabolomics approach to identify metabolic changes associated with NAFLD in humans upon high-intensity interval training (HIIT) across four different sample matrices—adipose tissue (AT), plasma, urine, and stool. This will enable understanding the complex nature of exercise-related benefits on health at the whole-body level combining the interplay of gut microbiota, circulation, excretion, and tissue metabolism.

## Results

### Clinical characteristics

**Figure 1:**
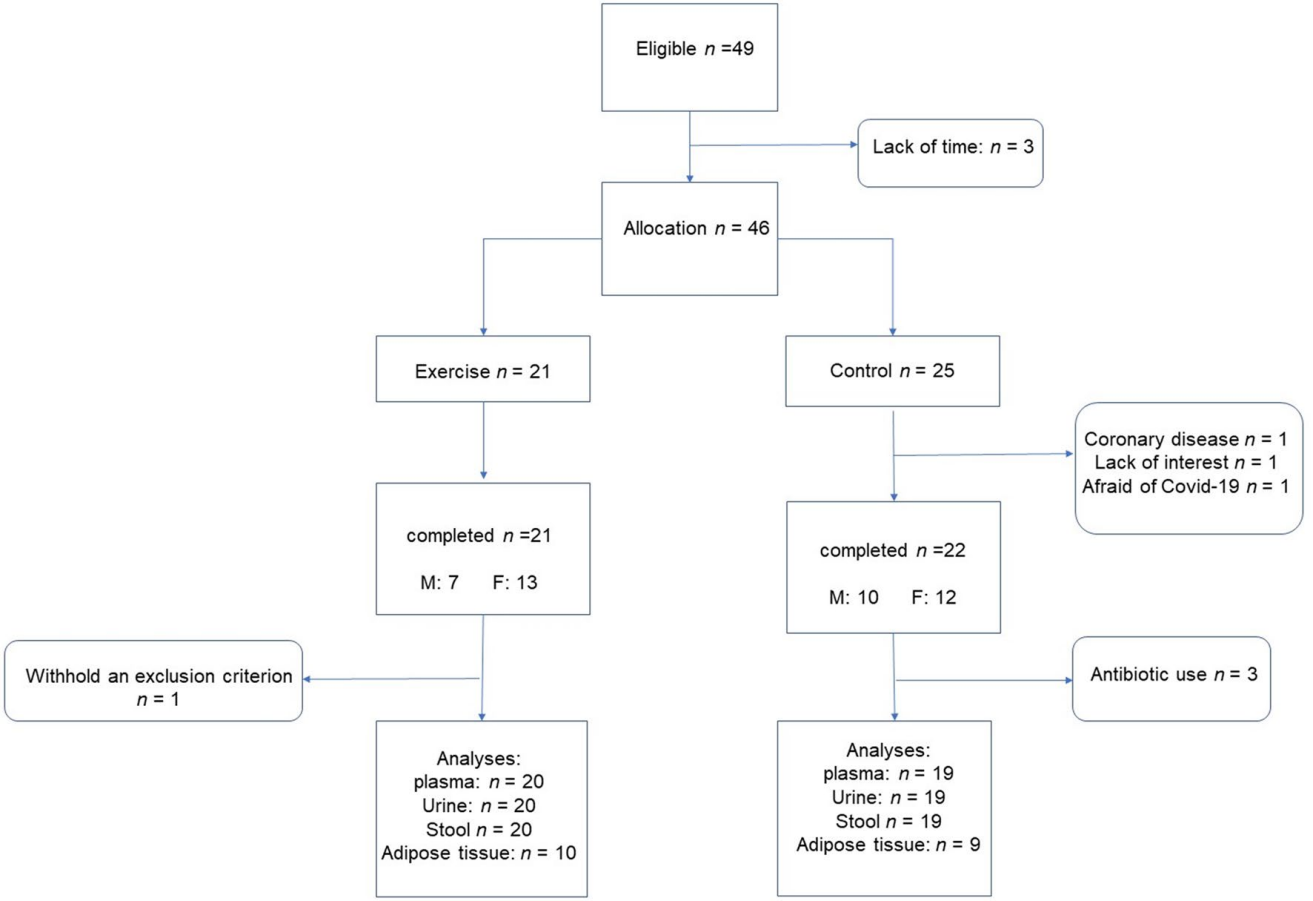
Flow chart of the study, *M* male, *F* female.

The subjects (n = 46) were randomly assigned into two study groups (intervention n = 21; control n = 25) according to the medians of body mass index (BMI), age, sex, and T2D status. The intervention group performed HIIT on an ergometer for 12 weeks while the control group kept their sedentary lifestyle. During this study, three people from the control group dropped out because of suspected coronary disease, lack of interest, or fear due to the Covid-19 pandemic. In the intervention group, one subject was excluded after finishing the study due to concealed use of injected diabetes medication, which was an exclusion criterion. In the control group, three subjects used antibiotics during the intervention, and were excluded in the metabolomics analysis. In total, 42 subjects finished the study (Fig. 1), and metabolomics analysis was conducted on samples from 39 subjects. Owing to the Covid-19 pandemic, adipose tissue biopsies at week 12 could not be performed from March 2020 onwards due to Finnish national regulations. Therefore, 19 adipose tissue biopsy samples from baseline and endpoint were available and further analyzed.

Anthropometric and clinical parameter results are shown in Table 1. For clinical parameters, 22 subjects in the control group and 20 subjects in the intervention group were analyzed. The mean age and BMI in both groups were similar (Table 1). Two subjects in the control group and three in the intervention group had T2D. The groups did not differ in body weight, BMI, or blood pressure at baseline or the end of the study. Waist circumference decreased significantly in the intervention group compared to the control group after 12 weeks (*p* = 0.028). Body weight stayed stable in both groups.

**Table 1:** Clinical parameters of the study.

Characteristics	Control group n = 22		Intervention group n = 20		Between group comparison based on LR^1^
	Week 0	Week 12	Week 0	Week 12	p-value
Age, yr	56.7 ± 10.7		59.9 ± 9.8		
Sex, M/F	10/12		7/13		
T2D patients	2		3		
BMI, kg/m^2^	29.5 ± 4.3	29.6 ± 4.3	29.7 ± 3.2	29.7 ± 3.2	0.491
Weight, kg	86.5 ± 16.0	86.6 ± 15.9	83.8 ± 15.6	84.0 ± 16.0	0.568
Waist cf, cm	101.6 ± 12.1	102.5 ± 11.9	101.2 ± 11.4	100.5 ± 12.2	**0.028**
Systole, mmHg	133 ± 20	128 ± 15	139 ± 12	137 ± 11	0.12
Diastole, mmHg	89 ± 10	88 ± 10	89 ± 7	88 ± 6	0.89
IHL, %	15.08 ± 11.0	14.18 ± 10.0	16.08 ± 9.11	14.94 ± 9.35	0.698
ALT, U/L	56.3 ± 33.0	58.0 ± 43.7	50.0 ± 21.8	46.6 ± 21.8	0.664
AST, U/L	36.9 ± 10.2	37.6 ± 17.3	33.0 ± 9.8	31.2 ± 10.3	0.826
y-GT, U/L	83.4 ± 90.6	86.0 ± 103.0	77.7 ± 70.5	83.9 ± 98.1	0.754
FLI, %	72.0 ± 22.7	71.3 ± 24.3	71.4 ± 24.6	70.7 ± 26.2	0.945^2^
HSI	42.5 ± 6.5	42.2 ± 5.9	43.2 ± 4.8	42.5 ± 4.3	0.350^3^
TC, mmol/L	4.9 ± 1.0	4.8 ± 0.9	4.7 ± 1.1	4.5 ± 1.2	0.683
HDL-C, mmol/L	1.38 ± 0.37	1.34 ± 0.34	1.44 ± 0.39	1.46 ± 0.42	0.137
LDL-C, mmol/L	3.2 ± 0.9	3.1 ± 0.8	3.0 ± 1.2	2.7 ± 1.1	0.549
TG, mmol/L	1.78 ± 0.98	1.84 ± 1.12	1.71 ± 0.78	1.67 ± 0.77	0.351
TC/HDL-C ratio	3.8 ± 1.2	3.8 ± 1.1	3.5 ± 1.1	3.3 ± 1.1	0.202
Apo A1, g/L	1.49 ± 0.22	1.45 ± 0.19	1.53 ± 0.2	1.55 ± 0.22	0.069
Apo B, g/L	1.04 ± 0.19	1.03 ± 0.3	0.97 ± 0.29	0.92 ± 0.3	0.283
HbA_1C_ mmol/L	37.5 ± 2.8	38.1 ± 2.8	40.5 ± 5.5	40.6 ± 5.3	0.502
Glucose, mmol/L	5.9 ± 0.6	6.1 ± 0.7	6.5 ± 0.9	6.3 ± 0.7	**0.027**
Insulin, mU/L	20.0 ± 2.2	21 ± 2.3	17.8 ± 3.1	17.0 ± 1.9	0.370
hs-CRP, mg/L	2.0 ± 1.3	2.4 ± 3.1	1.2 ± 1.0	2.5 ± 3.1	0.513
VO_2_max*, mL/min	2159 ± 543	2134 ± 493	1990 ± 561	2162 ± 631	**< 0.001**
VO_2_max*, mL/kg/min	25.1 ± 5.3	24.9 ± 4.8	23.7 ± 4.02	25.7 ± 4.4	**< 0.001**
maxW*, watt	165.6 ± 57.5	163.5 ± 57.2	150.0 ± 51.7	168.3 ± 50.1	** < 0.001**

*yr* year, *M* male, *F* female, *T2D* type 2 diabetes, *BMI* body mass index, *cf* circumference, *ALT* alanine transferase, *AST* asparagine transferase, *y-GT* gamma-glutamyl transferase, *HSI*—hepatic steatosis index, *FLI* fatty liver index, *TC* total cholesterol, *HDL-C* high-density lipoprotein cholesterol, *LDL-C* low-density lipoprotein cholesterol, *TG* triglyceride, *HbA*_*1C*_ glycated haemoglobin, *hs-CRP* high-sensitive C-reactive protein, *VO*_*2*_*max* maximum rate of oxygen consumption = “cardiorespiratory fitness”, *maxW* maximum workload achieved; *based on 19 exercise test; ^1^Linear regression model (Covariates: T2D, gender, age, BMI); ^2^Linear regression model (Covariates: T2D, gender, age); ^3^Linear regression model (Covariates: age); Values are means ± SD. Significant values are in bold.

A significant decrease in fasting plasma glucose concentration (*p* = 0.027) was observed between the control and intervention groups at 12 weeks (Table 1). In addition, glucose concentration and HbA1c were measured via Freestyle libre for 2 weeks before the study and during the two last weeks. Each subject measured the parameters several times per day via a device. No significant changes were found within or between the groups during the study. The intrahepatic liver content did not change significantly at the end of the study (*p* = 0.698), as well as the liver enzymes ALT, AST, and γ-GT in fasting plasma. Furthermore, in fasting plasma, alkaline phosphatase, albumin, and bilirubin showed no changes (data not shown). In addition, no significant changes in the lipid parameters in fasting plasma were observed. The hepatic steatosis index (HSI) and the fatty liver index (FLI) were also not significantly changed.

The exercise parameters, cardiorespiratory fitness (maximum rate of oxygen consumption, (VO_2_max), and maximum achieved workload (maxW) were analyzed from 22 subjects in the control group and 19 subjects in the intervention group. One person in the exercise group did not participate in the final ergospirometry test at week 12. Fitness levels expressed as VO_2_max and maxW improved in the HIIT group and differed significantly from the control group (*p* < 0.001). No significant changes in the concentration of an inflammation marker high-sensitive C-reactive protein (hs-CRP) were found.

The habitual diet was to be kept unchanged during the intervention, and there were no differences within or between the groups during the study (Supplementary Table 1). In addition, no changes were observed in the body composition (Supplementary Table 2).

### Metabolite profiling

**Figure 2:**
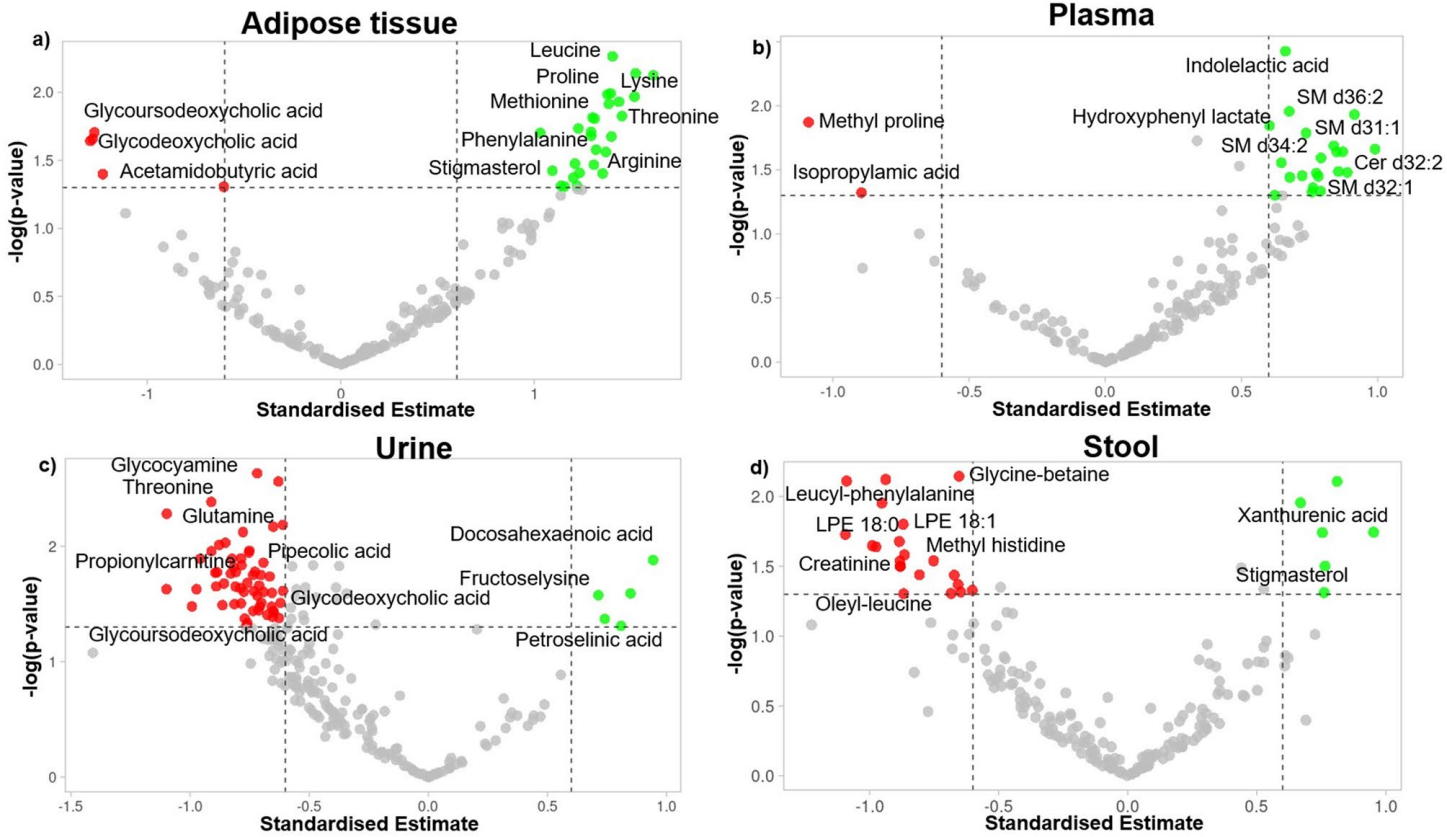
Volcano plot of significantly different metabolites from the exercise intervention in (**a**) adipose tissue, (**b**) plasma, (**c**) urine, (**d**) stool. The data of all metabolites are plotted as the standardized estimates of the linear mixed model for the interaction between group (controls and intervention) and time (baseline and endpoint of the exercise intervention) versus the negative logarithm of the raw p-values. Thresholds are shown as dashed lines. Metabolites selected as significantly increased in the intervention group are highlighted as green dots, and those decreased significantly in the intervention group compared to the controls are shown as green dots.

The non-targeted LC–MS analysis of the metabolic profiles showed differences between the four different sample matrices—plasma, adipose tissue (AT), urine, and stool, as visualized by principal component analysis (Supplementary Fig. 1). The plasma and AT samples were most similar to each other. The HIIT intervention caused marked differences in the metabolite levels across all the examined sample matrices (Fig. 2). However, the metabolic signature in each of the sample types was different, with varying compositions of metabolites either increasing or decreasing upon the HIIT intervention. As detailed below, the most notable differences were found in amino acids and their derivatives, lipids, and bile acids.

### Amino acids and their derivatives

When comparing the effect of the intervention between the intervention and control group, the most notable group of differential compounds was amino acids and their derivatives, including metabolites of amino acids and di- and tripeptides, as visualized in the volcano plot and the pathway analysis (Fig. 2 and Supplementary Fig. 2). Most of these compounds increased in particular in the AT as well as plasma in the intervention group, while they decreased in the urine and stool samples (Fig. 2, Supplementary Table 3). Notably, the proteinogenic amino acids (arginine, leucine, lysine, phenylalanine, methionine, tyrosine, threonine, and proline) increased in AT in the intervention group. In addition, other derivatives of these amino acids such as leucic acid (leucine metabolite), acetyllysine, dimethyllysine, proline-hydroxyproline, and dimethylarginine were also similarly increased in the AT in the intervention group. The most decreased metabolite in plasma after the exercise intervention was methylproline, whereas dimethylarginine, tryptophan metabolites (indolelactic acid and 3-(4-hydroxyphenyl)lactate), alanine derivative (sulfoxyphenylacetyl dehydroalanine), noradrenaline metabolite ((3-methoxy-4-hydroxyphenyl)ethylene glycol sulfate), and glutaric acid were all increased in the plasma due to the HIIT intervention. In the urine samples, amino acids such as glutamine, ornithine, and threonine; and the derivatives such as methylindole, methylcrotonylglycine, gamma-glutamylleucine, trimethyllysine, and isatin decreased in the intervention group. Further, a lysine derivative (fructoselysine) and a methionine derivative (adenosyl-methionine) increased in the intervention group. In the stool samples, several leucine containing peptides (leucyl-leucine, threonyl-leucine, oleyl-leucine, leucyl-phenylalanine) and other glycine and histidine derivatives such as glycocyamine, glycine-betaine, and methylhistidine decreased in the intervention group, while xanthurenic acid, a metabolite in the kynurenine pathway of tryptophan metabolism, increased. Consistent with these findings, when the data were analyzed using MetaboAnalyst^18^, several amino acid metabolism pathways in AT, urine, and stool were changed markedly (Supplementary Fig. 2).

**Figure 3:**
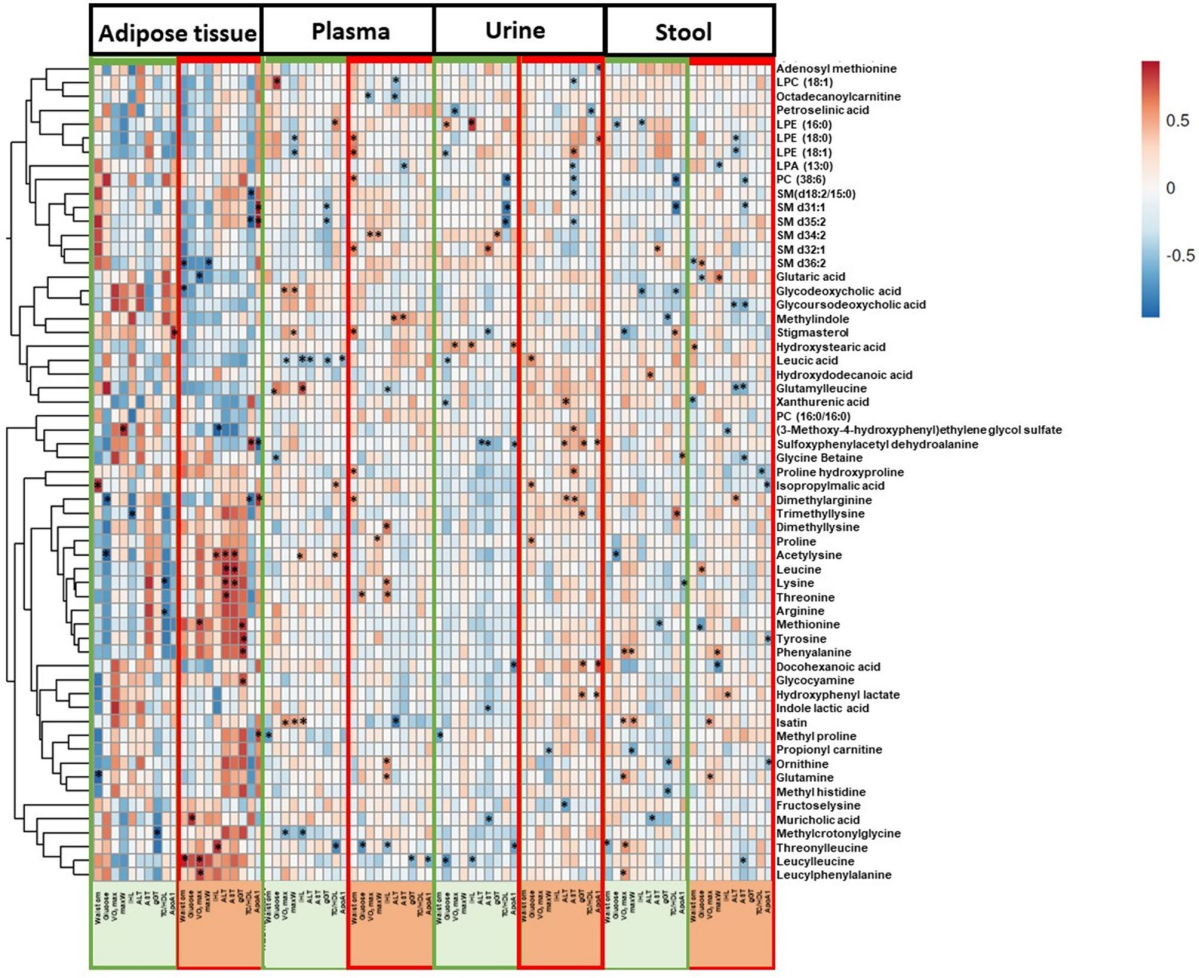
Heatmap representing the significant Spearman's correlations (adjusted for age, gender, BMI, and T2D) between the clinical parameters (column-wise) and the top significantly different metabolites identified in all sample matrices combined (row-wise) using delta change from baseline to post intervention. The color of the cells indicates the strength of the relationship (r_s_). The cells marked with asterisks (*) demonstrate significant correlations (p < 0.05). Green sidebars indicate the control group, and red sidebars represent the exercise intervention group. *Waist cm* waist circumference, *IHL* intrahepatic lipid content, *ALT* alanine aminotransferase, *AST* aspartate transaminase, *gGT* gamma-glutamyl transferase, *VO*_*2*_*max* the maximum rate of oxygen consumption (cardiorespiratory fitness), *maxW* maximum workload achieved, *TC* total cholesterol, *HDL* high-density lipoprotein cholesterol.

The differential metabolites were also correlated with various clinical parameters to further examine their relevance to NAFLD and exercise. The changes of the improved clinical parameters including fasting plasma glucose concentration, VO_2_max, and waist circumference were mostly positively correlated with amino acids in AT in the intervention group (Fig. 3). In the intervention group, waist circumference was positively correlated with leucyl-leucine in AT; dimethylarginine, methylproline, and proline hydroxyproline in plasma; and negatively correlated with xanthurenic acid in stool. Plasma glucose concentration was positively correlated with threonine in plasma; proline, leucic acid, and isopropylmalic acid in urine; and leucine in stool. Significant negative correlations of trimethyllysine in plasma; and glutaric acid and methionine in stool with waist circumference were also observed. VO_2_max was significantly positively correlated with leucyl-leucine, leucylphenylalanine, and methionine in AT; and glutamine and isatin in stool. Proline in plasma; and glutaric acid and phenylalanine in stool positively correlated with maxW. In AT, while strong positive correlations of most of the amino acids and derivatives were observed with the liver parameters (Fig. 3), only proline, methylproline, sulfoxyphenylacetyl dehydroalanine, and isatin correlated significantly with lipid parameters (Fig. 3 and Supplementary Fig. 3).

### Lipids and lipid-derived compounds

Exercise also induced changes in several lipids and their derived compounds, including sphingolipids, bile acids, glycerophospholipids, acylcarnitines, and sterols across the four different sample matrices. Moreover, the pathway analysis also revealed marked differences in lipid metabolism pathways in plasma (Supplementary Fig. 2). As visualized in the volcano plot (Fig. 2), in plasma, six sphingomyelins (SMs) [SM d31:1, SM d32:1, SM d34:2, SM d35:2, SM d36:2, SM d(18:2/15:0)] and a ceramide (Cer d32:1) increased in the intervention group. In the intervention group, these SMs were positively correlated with waist circumference, plasma glucose concentration, VO_2_max, and maxW in plasma (Fig. 3). Significant positive correlations of SM d32:1 with waist circumference; and SM d34:2 with VO_2_max and maxW were observed. In AT and urine, bile acids were found to be decreased. As an example, glyco-conjugated bile acids such as glycoursodeoxycholic acid and glycodeoxycholic acid decreased in AT; glycoursodeoxycholic acid and muricholic acid decreased in urine. These bile acids were negatively correlated with waist circumference, glucose, VO_2_max, maxW in AT while positively correlated in plasma (Fig. 3).

Further, in the intervention group, an increase of several glycerophospholipids in AT and plasma and a decrease in the stool (Fig. 2, Supplementary Table 3) were found. Notably, LPE 18:0 and LPE 16:0 increased in AT. In addition, LPE 16:0, PC 38:6, PC 32:0, and LPA 13:0 increased in plasma. A significant positive correlation of PC 38:6 with waist circumference; negative correlation of LPC 18:1 with ALT; and negative correlation of LPA 13:0 with AST was observed. LPC 18:1, LPE 18:0, and LPE 18:1 decreased in the intervention group in stool samples. Here, LPE 18:0 was negatively correlated with ALT in the intervention group in the intervention group.

In the intervention group, sterols such as stigmasterol increased in AT and stool. Moreover, fatty acids also increased in AT, plasma, and urine. As an example, 12-hydroxydodecanoic acid (medium chain fatty acid) increased in AT, 2-hydroxystearic acid (long-chain fatty acid) increased in plasma, and docosahexaenoic acid (very long chain fatty acid) and petroselinic acid (long-chain fatty acid) increased in urine. Moreover, acylcarnitines (propionylcarnitine and octadecanoylcarnitine), which are also fatty acids bound to carnitine, decreased in urine and stool respectively in the intervention group. In the intervention group, stigmasterol in stool was negatively correlated with TG and ApoB. In AT stigmasterol correlated negatively with TC and LDL-C concentrations (Supplementary Fig. 3). Docosahexaenoic acid in urine was positively correlated with ApoA1, ApoB, and TC in the intervention group (Fig. 3 and Supplementary Fig. 3). Octadecanoylcarnitine in urine was positively correlated with plasma insulin concentrations and negatively correlated with LDL-C concentration in the stool in the intervention group (Supplementary Fig. 3).

Further, the significantly improved clinical parameters including waist circumference, VO_2_max, and fasting glucose concentration correlated with many metabolites in the intervention group (Fig. 3). These included negative correlations of waist circumference and glucose concentration with lipids (including bile acids) in AT; but positive correlations with waist circumference and plasma glucose concentrations. In addition, sphingomyelins were positively correlated with waist circumference, VO_2_max, and maxW in plasma.

## Discussion

Physical exercise offers benefits for NAFLD patients^19^. However, the underlying mechanisms by which exercise modulates these effects remain poorly understood. In this study, a non-targeted metabolomics approach was employed to elucidate these mechanisms by identifying metabolic changes associated with NAFLD and its related clinical parameters upon an exercise intervention.

The exercise intervention was successful and resulted in significant decrease in plasma glucose concentration and WC and increase in exercise parameters (VO_2_max and maxW) without weight loss and dietary changes. The drop-out rate of the whole study was low, and 18 out of 19 subjects finished more than 20 of the 24 supervised exercise sessions. The exercise parameters VO_2_max and maxW were significantly increased compared to the control group at the end of the intervention. In line with our study, several other exercise studies have shown, fitness levels increase with continued training independently of the exercise regime^20–22^. Further, increased VO_2_max levels are known to confer protection against hyperglycemia and cardiovascular disease mortality^23,24^. The drop-out rate of the whole study was low, and 18 out of 19 subjects finished more than 20 of the 24 supervised exercise sessions. The high compliance might be explained by well-designed individual training plans based on the subject’s fitness at baseline.

Fasting plasma glucose concentration was significantly decreased, whereas insulin concentration and HOMA IR (data not shown) did not decrease significantly in the intervention group compared to the control group. The improved glucose homeostasis after exercise can be partly explained by enhanced glucose uptake in skeletal muscles via the transport protein GLUT4, leading to decreased plasma glucose concentration^25^. A non-significant result in insulin concentration can be explained by high variability as well as by the intended absence of weight loss. WC decreased in the exercise group after the intervention compared to the control group. WC is an essential indicator of central obesity and insulin resistance, and it is strongly associated with cardiovascular mortality but also all-cause mortality^26,27^. A decrease in WC can reduce these risks and the risk of developing T2D^28^. However, the liver fat did not significantly decrease during the intervention compared to the control group. Other exercise interventions have resulted in liver fat reduction without weight loss^29,30^.A potential explanation is that the liver adiposity was not high in the subjects participating in this study. Although they had NAFLD diagnosis, some of them were in a good control and this is why their liver adiposity was within normal range. Another explanation could be the number of exercise sessions per week. While Winn et al*.* and Johnson et al*.* conducted four and three supervised HIIT sessions per week^29,30^ respectively, we had two supervised sessions per week, which might not be enough to show significant results with this number of subjects. Further, no improvement in plasma lipid profile was observed. This is likely due to the lack of dietary modification. A common Finnish diet is high in saturated fat^31^ and the subjects were instructed to keep their dietary habits unchanged during the study.

Our metabolomics investigation combined with the pathway analysis of the identified metabolites showed significant changes especially in amino acids and their derivatives after the exercise intervention (Fig. 2, Supplementary Fig. 2, Supplementary Table 3). An accumulation of amino acids in AT was observed in the intervention group. Particularly interesting was the increase of branched chain amino acid (BCAA) leucine. In obese and insulin-resistant people, BCAAs are known to have reduced levels in AT and elevated concentrations in plasma^32,33^. This causes massive decomposition of these amino acids in skeletal muscle and liver, and induces insulin resistance, thereby causing NAFLD progression. However, the observation of accumulated BCAAs in AT in the intervention group could imply the enhancement of mitochondrial oxidative potential of BCAAs upon exercise, which could alleviate or eliminate the accumulation of toxic catabolic intermediates of BCAAs that induce insulin resistance^32^. Other beneficial effects of increased BCAAs, in adipocytes, especially leucine, might be mediated by the activation of the mechanistic target of rapamycin (mTOR), resulting in AT morphogenesis, differentiation of adipocytes, hyperplastic growth, leptin secretion in adipocytes, and thermogenesis^34–36^. In addition, BCAAs partly play a role in mitochondrial biogenesis in skeletal muscle cells and adipocytes, which modulates the lipid metabolism as inhibition of fat synthesis and increased fatty acid oxidation^37–39^. Leucine may also promote the browning of the AT via this pathway, but the results are contradictory, and further studies are warranted^40^.

Besides BCAAs, other amino acids such as methionine, threonine, and tyrosine increased in AT in the intervention group. These amino acids could also contribute to enhanced lipid and glucose metabolism via the activation of the PGC-1α1-PPARα/ɣ signaling cascade. As an example, dietary supplementation of threonine improves lipid metabolism in obese mice by PPAR-γ signaling cascade along with the down-regulation of lipogenesis expression levels and up-regulating of lipolysis expression levels^41^. Physical exercise also promotes the activation of the PPAR-γ/PGC-1α axis. This causes irisin release, thereby promoting the browning of beige fat cells in white adipose tissue, enhanced thermogenesis, increased energy expenditure, and maintenance of glucose homeostasis^42,43^. The increase of threonine levels in vivo in the intervention group could be partially responsible for these beneficial effects; however, further studies are warranted^44^. Similarly, the increase of tyrosine levels as observed from the metabolomic and pathway analysis (Fig. 2 and Supplementary Fig. 2) could also contribute towards the exercise-related benefits. The hydroxylation of phenylalanine results in the formation of tyrosine. Tyrosine could then be used as a catecholamine precursor, which plays a vital role in improving athletic performance and regulates lipolysis in AT^45,46^. Therefore, the reduced levels of phenylalanine compared to tyrosine could imply increased tyrosine production to account for the need for catecholamines during exercise^47^.

In contrast to the accumulation of amino acids in AT, their levels decreased in urine during the intervention. Amino acids are generally higher in the urine samples of NASH patients than people without NASH due to the abnormalities in the amino acid metabolism^16^. A decrease of amino acids and their derivatives in urine found in this study could imply lesser hepatic damage and lower release of amino acids from the liver. The reduced presence of peptides in the stool could imply an increased gut microbiota diversity, associated with exercise and could promote an improved protein degradation^48^. The produced branched-chain fatty acids (BCFA) and short chain fatty acids (SCFA) can modulate glucose and lipid metabolism^49–51^. In addition, propionylcarnitine was increased in plasma. It is an SCFA esterified to carnitine and diffuses through the serosal membrane into the circulatory system to reach mainly skeletal and cardiac cells^52,53^. Therefore, it could be a potential marker for SCFA production. Its bioavailability is better than carnitine, and it can ameliorate cardiovascular dysfunction and metabolic disorders, including insulin resistance^54^.

The most significantly decreased metabolite in plasma after the exercise intervention was the AA derivative methylproline, which is a biomarker of fibrosis^55^. Mardinoglu et al*.,* previously reported a correlation of methylproline with hepatic steatosis in the plasma samples. Also, plasma methylproline levels were significantly changed between subjects with high and low steatosis^56^. In addition, methylproline is a gut microbiota produced metabolite^57^. Therefore, we propose that exercise-induced change in gut microbial composition or function could alleviate NAFLD by decreasing the levels of this compound. However, more studies are warranted.

The observed change in several gut microbiota -borne metabolites such as indolelactic acid, indicates that either the composition or function of gut microbiota was altered due to the intervention. Indolelactic acid acts as a ligand for the aryl hydrocarbon receptor (AhR) and is expressed by immune cells that regulate intestinal immune homeostasis^58^. The activation of AhR promotes the production of interleukin-22, thereby stimulating mucosal defense by inducing antimicrobial proteins^58^. Moreover, AhR activation also decreases glucose and triglyceride levels in serums of subjects with obesity and metabolic syndrome^59^. Thus, an increase of indolelactic acid in the intervention group's plasma could alleviate NAFLD by enhancing the stimulation of mucosal defense, modulating inflammatory responses, and glucose homeostasis in an AhR-dependent manner. Similar observations of increased indole lactic acid levels in plasma samples have been reported after a maximal exercise cycling test^60^.

Perturbations in lipid metabolism contribute towards NAFLD development^61^. Specific species of these lipids, such as glycerophospholipids and sphingolipids, have been involved in oxidative stress, insulin resistance, inflammation, and cell death^61,62^. SMs are the most abundant sphingolipids and are known to regulate insulin and glucose homeostasis^63,64^. In particular, very long-chain sphingolipid species (C < 22) are known to protect against glucose intolerance and hepatic insulin resistance development^65^. In this study, very long chain SMs including SM d31:1, SM d32:1, SM d34:2, SM d35:2, SM d36:2, SM d(18:2/15:0) increased in plasma in the intervention group, further highlighting the potential of these increased SMs in terms of improved insulin/glucose homeostasis. However, due to the diversity of SM species, some are also known to be mediators of insulin resistance and could disturb glucose homeostasis^63,64^. Further, altered levels of SMs are implicated in the development and severity of NAFLD^14,61,66^, however, the results have been inconsistent. As an example, increased SMs in the plasma samples of obese children with hepatic steatosis have been reported by Draijer et al.^67^. On the contrary, Zhou et al. reported decreased levels of SMs in NASH patients compared to their non-NASH counterparts^66^. In addition, the results of exercise intervention studies related to the SMs have been inconsistent. While one study found no differences in serum sphingolipids after exercise^64^, another study showed significantly decreased concentrations of plasma ceramides (C14:0, C16:0, C18:1, and C24:0) and improved insulin sensitivity after exercise^68^. Although this could be explained by the diversity of SMs, further studies are warranted to elucidate the role of individual SM compounds in exercise.

Physical exercise also modulates lipid metabolism by inducing tissue-specific alterations^69^. Significant increases in phosphatidylethanolamines (PEs) and a significant decrease in phosphatidylcholines (PCs), lyophosphatidylcholines (LPCs), and lyophosphatidylethanolamines (LPEs) have been reported in obese children with steatosis^67^. In this study, we saw an accumulation of phospholipids in AT and plasma and their decrease in stool in the intervention group, which were also elaborated. We also observed marked changes in glycerophospholipid metabolism in the pathway analysis (Supplementary Fig. 2). Similar observations of increased hepatic lysophospholipids as a result of physical exercise were reported by Hoene et al.^69^. Li et al. who conducted a similar intervention study as ours, found that in the serum samples, the changes in PE, phosphatidylinositol (PI), and phosphatidic acid (PA) contents were contrary to our results in plasma^63^. This could be because these lipid species are heterogeneous and can have different effects related to fatty acyl chain composition^63^.

Bile acids are essential players of human metabolism implicated in the development and progression of NAFLD^70^. Elevated levels of total bile acid in serum, plasma, urine, liver, and stool have been previously reported in NAFLD and NASH subjects^13,71–73^. However, there are uncertainties regarding the nature and amount of change in bile acids in NAFLD^74^. Although not extensively studied in NAFLD subjects, exercise, in general, has been reported to decrease bile acid concentrations in the blood and stool^75–78^. Our study observed a decrease of bile acids in AT and urine. In the plasma and stool samples, this decrease was nominal. Although the observation of decreased bile acids after the intervention is well in line with earlier studies with a similar setup^75–77^, it is difficult to suggest why they were decreased in all sample matrices in our current study. Possible mechanisms are related to the fact that exercise likely reduces the amount of lipids, and this could, in turn, reduce bile acid synthesis while increasing bile acid absorption efficiency. This could cause lower amounts of bile acids to be excreted in the feces^78,79^. However, the observed lower level in AT and urine as presented here has not been demonstrated before. More studies are warranted to elucidate mechanisms across different sample matrices.

Increased levels of secondary bile acids produced by the gut microbiota are known to contribute towards NASH progression^70^. This study observed a decrease in secondary bile acids such as glycoursodeoxycholic acid and glycodeoxycholic acid. Similar observations of reduced glycoursodeoxycholic acid after endurance and resistance exercise and reduced glycodeoxycholic acid levels after resistance exercise were reported by Morville et al*.*^76^. Exercise, therefore, likely alters gut microbiota by decreasing glycine metabolizing bacteria, thereby reducing the levels of these glyco-conjugated bile acids. Moreover, decreased glycine levels after a bout of exercise in urine and blood were reported^75^. Similar observations of reduced (but non-significant) glycine levels were also found in our study (Supplementary Fig. 4). Therefore, the limited availability of glycine for bile acid conjugation could also be a reason for the observed decrease in glyco-conjugated bile acid in this study.

Reduced carnitine concentrations, in addition to causing cell oxidative damage, fatty acid synthesis, and energy metabolism disorders, often result in NAFLD^16^. Exercise, however, is known to accelerate fat metabolism, thereby increasing acylcarnitine levels in plasma^69,80^. Thus, the decrease of these acylcarnitines in urine and stool in this study might imply that they were used in circulation or as metabolic substrates for muscle activity^81^. This was further confirmed by the nominal increase of acylcarnitines in plasma.

Interestingly, stigmasterol, a plant-derived sterol, was increased in AT and stool after exercise. Stigmasterol is widely present in plant oil and plant-based foods and is known to have anti-osteoarthritic, anti-mutagenic, anti-inflammatory activity, and anti-tumor properties^82–85^. Since the diet did not change during the intervention, these changes in stigmasterol could be a result of the increased absorption following the exercise intervention.

Altogether, in this study, the exercise training induced improvements in waist circumference, fasting glucose concentration, VO_2_max, and maxW. The changes in these clinical parameters were strongly correlated with changes in metabolites during the exercise training. However, more studies are warranted to examine the relevance of these correlations in the context of effects of exercise in NAFLD. Furthermore, the changes in the identified metabolites during the intervention were mainly significantly correlated with the changes in the liver parameters rather than with lipid and glucose parameters. Especially in AT, stronger positive correlations of amino acids and their derivatives were observed with IHL, ALT, AST, γ-GT, particularly in the intervention group. While γ-GT was elevated and worsened non-significantly, all other clinical parameters improved nominally in the intervention group after 12 weeks. Nevertheless, it suggests that adipose tissue metabolism seems to have an essential role in the relationship between glucose homeostasis and exercise without weight loss and might play a crucial role in NAFLD improvement. Moreover, in plasma, several metabolites, especially SMs which have a central role in glucose and lipid metabolism, were positively correlated with significantly increased VO_2_max and/or maxW. Very long-chain sphingolipid species (C < 22) have protective effects against glucose intolerance and hepatic insulin resistance development^65^. Moreover, some long-chain sphingolipids have been reported to be useful markers of fitness and response to exercise in coronary artery disease^86^; however, their role in NAFLD is poorly understood. Therefore, the positive correlation of SMs with fitness parameters could possibly indicate an improvement of glucose and lipid metabolism in NAFLD with increased exercise intensity. In addition, the lipid metabolites including bile acids were altered independent of classic markers of lipid metabolism, i.e. concentrations of LDL-C, HDL-C, triglycerides, and total cholesterol. This could possibly mean that the relative risk reduction in NAFLD and CVD with exercise training is not limited to classic markers of cardiometabolic health. This might further support the potential additive benefits of exercise to various drug therapies.

**Figure 4:**
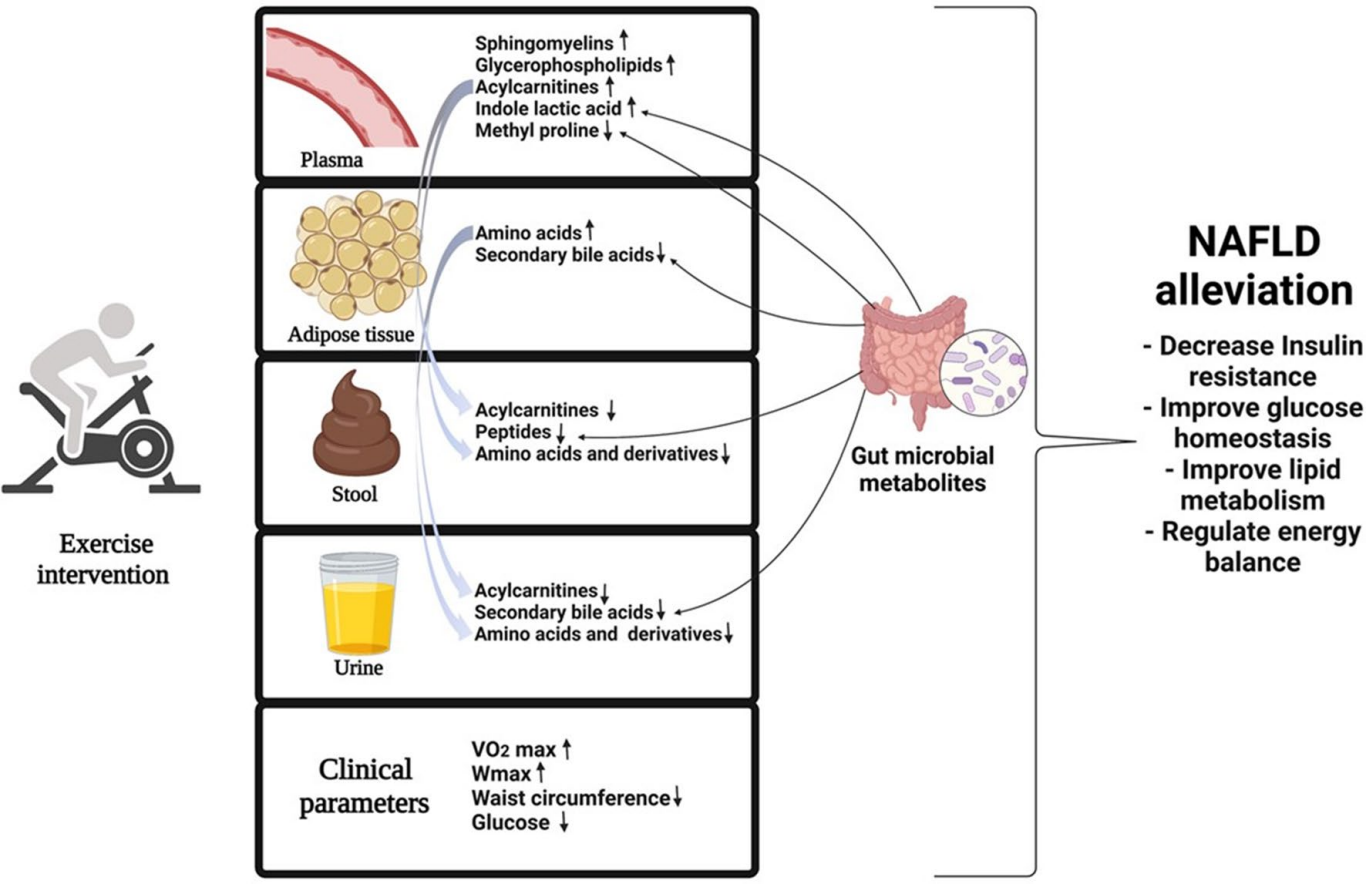
A 12-week high-intensity interval training has beneficial ameliorating effects in NAFLD subjects at whole body level by regulating glucose metabolism and promoting alterations in amino acid, lipid, and bile acid metabolism.

Several studies have highlighted that exercise benefits patients with NAFLD, however, the metabolites, mechanisms, and pathways still remain poorly understood. To the best of our knowledge, this study was the first to study the metabolic changes in NAFLD subjects due to physical exercise across various sample matrices. In addition, we provided a comprehensive metabolic readout across four different sample matrices and highlighted that the altered amino acid and lipid metabolism might contribute to the mechanisms underlying the beneficial effects of exercise in NAFLD patients. Notably, as seen in Fig. 4, the majority of the metabolite alterations were sample type-specific, highlighting the importance of whole-body metabolic homeostasis and interplay with gut microbiota, circulation, excretion, and tissue metabolism, which cannot be studied if focusing on single sample specimens. This study showed that a 12-week HIIT exercise intervention has beneficial ameliorating effects in NAFLD subjects on a whole-body level, even without dietary changes. Even without significant weight loss, HIIT decreased plasma glucose concentration and waist circumference, which is an essential indicator of central obesity and is associated with insulin resistance. Further, HIIT increased exercise parameters (VO_2_max and maxW), which are known to protect against hyperglycemia. The decreased plasma glucose concentration was also implicated at the metabolite level, wherein concentration of plasma SMs, which are involved in regulating glucose homeostasis, were increased. The observed accumulations of amino acids in plasma and AT; and decreased urinary and fecal excretion in the intervention group could further indicate their improved utilization and glucose metabolism. In addition, our study provided several hints of exercise mediating metabolic benefits by inducing changes in the gut microbial composition or function, including SCFAs and indole lactic acid which are involved in regulating energy and glucose balances.

Although we profiled a wide range of metabolites across various sample matrices in NAFLD subjects upon exercise, further validation in larger studies would strengthen the observed results. Moreover, complementing this study with comprehensive genomic and gene expression analysis could help in further exploration of the mechanisms behind NAFLD improvement. Additionally, animal models could be employed to examine the relevance of the correlations observed in our study. Furthermore, metabolic profiling of muscle and liver tissue might help further advance our knowledge about exercise-induced inter-organ crosstalk in NAFLD. Moreover, the metabolomics analysis is still limited because of the incomplete availability of MS/MS spectral libraries for annotation of all the significant metabolites, resulting in a relatively large number of features that could not be annotated, warranting future studies to investigate the changes occurring in these compounds along with providing confirmation to the identifications. The study had, however, limited number of subjects. Also, the oral glucose tolerance test, providing a body's response to a glucose load, could not be performed due to practical issues related to very binding schedule to the participants. Overall, our results indicate that AT might play a critical role in improving plasma glucose concentration and mediating exercise-related benefits. Further, without significant weight loss and dietary changes, exercise might ameliorate NAFLD conditions by regulating glucose metabolism and promoting alterations in amino acid, lipid, and bile acid metabolism upon exercise.

## Methods

### Study

This study was a randomized, controlled exercise intervention study. The randomization was conducted by the study nurse based on a randomization table by matching the subjects according to BMI, age, gender, and T2D status in two groups (Fig. 1). The intervention group, the high-intensity interval training group (HIIT group), followed a 12-week exercise intervention which was prescribed individually based on the ergospirometry test. The control group maintained their sedentary lifestyle with no changes in physical activity. Subjects completed three scheduled study visits at baseline, week 6, and week 12. The trial finished in June 2020.

The primary outcome was IHL, and the secondary outcomes were liver enzymes, glucose, and lipid parameters.

### Participants

Altogether 49 subjects diagnosed with NAFLD were recruited from the Kuopio University Hospital (KUH), Kuopio Health Care Centre, and Occupational Health Care. In total, 42 subjects finished the study (Fig. 1). The recruitment started in April 2019 and ended in September 2019. The trial finished in June 2020.

The inclusion criteria were age 18–70 years and BMI below 35 kg/m^2^. The NAFLD diagnosis was based on the liver ultrasound examination, magnetic resonance imaging, or computed tomography. Subjects using metformin, sulfonylureas, SGLT2 receptor inhibitors, statins, and/or blood pressure medication and subjects with stable hypothyroidism on thyroxin medication were eligible. The main exclusion criteria included acute illness or current evidence of acute or chronic inflammatory or infective diseases. No subjects with hepatitis B and/or C, autoimmune hepatitis, Wilson's disease/alpha-1-antitrypsin deficiency, hemochromatosis, unstable hypothyroidism, lipoatrophy, bleeding disorder, anticoagulant medication, or those not able or willing to undergo MRI (e.g., claustrophobia, implantable cardioverter defibrillator, pacemaker) were included. In addition, any neurological, musculoskeletal, or cardiorespiratory conditions, which would put the subjects at risk during exercise or inhibit their ability to adapt to an exercise program, were excluded. Furthermore, participation in a regular exercise and/or diet program within 3 months before recruitment was an exclusion criterion. No subjects with diagnosed type 1 diabetes or diagnosed T2D with insulin or GLP-1 agonist treatment were included. In addition to that, subjects diagnosed with depression or any mental illness rendering the subject unable to understand the nature, scope, or possible study sequences, were not accepted. Smoking and alcohol abuse (daily consumption ≥ 30 g for men and ≥ 20 g for women) were also exclusion criteria.

### Diet

The subjects were instructed to keep their dietary habits unchanged. They kept a 4-day food record just before the intervention period and at week 11 during the intervention. The food record was kept during predefined consecutive days (3 weekdays, 1 weekend day) and checked by a clinical nutritionist at return. The AivoDiet software (version 2.2.0.0, Aivo Finland Oy, Turku, Finland) was used to calculate the nutrient intakes.

### Exercise

#### Assessment of cardiorespiratory fitness

To determine the intensity of the individually prescribed training program and the efficacy of the exercise intervention, ergospirometry tests were carried out on a cycle ergometer (Ergoline, Bitz, Germany) at baseline and after 12 weeks for intervention and control groups. The test started with a 1-min sitting period in the saddle, followed by a 3-min warm-up with 0 Watts (W). After that, the workload was increased gradually at 6 s intervals according to an individualized protocol. The test was supervised by a physician, and participants were verbally encouraged to continue until exhaustion. Respiratory gas exchange and ventilation were measured by the breath-by-breath method, and electrocardiography (ECG) was recorded throughout the exercise test.

#### Exercise intervention

The exercise intervention started immediately after the ergospirometry test. The intervention group followed a HIIT protocol on a cycle ergometer^87^. In detail, the concept of HIIT involved repeated bouts of exercise at an intensity of 85% of maxW4 interspersed by recovery periods based on a baseline ergospirometry test (Supplementary Table 4). maxW4 referred to the hypothetical workload sustainable for 4 min^88^. While being hypothetical, maxW4 was useful for defining the presumably optimal intensity for work intervals at HI. The intervention group performed HIIT sessions involving five bouts of 2–4 min work intervals (at 85% of maxW4) interspersed by 3 min of active recovery (at 20% of maxW4) period twice per week on non-consecutive days for 12 weeks. Each work interval was 2 min long in the first week, with 5 s added per exercise session (i.e., 10 s per week) so that HIIT intervals were 4 min long by week 12. At the beginning of the intervention period, each HIIT session lasted approximately 40 min, including warm-up (at 30% of maxW4), recovery periods, and cool-down (at 20% of maxW4). At the end of the intervention period, each HIIT session lasted approximately 50 min. HIT was conducted in a group of 1–3 subjects closely supervised by an exercise physiologist or other qualified health care professional (e.g., biomedical laboratory scientist) adequately trained for the task. After the Covid-19 outbreak, the group size was reduced to 1–2 subjects for each session. Each subject's first six training sessions were followed through under ECG monitoring.

Additionally, an individualized exercise training program was prescribed for each subject in the HIIT group consisting of home-based low-to moderate-intensity aerobic exercise. An overall weekly goal, including supervised exercise, was 3 h of aerobic exercise following international guidelines^89^. Home-based exercise was, e.g., walking, swimming, and cycling. The control group was given usual care according to regional medical practice for NAFLD patients, and they were instructed to maintain their physical activity unchanged during the study.

Leisure-time physical activity at baseline and during the intervention period was assessed by the modified Minnesota Leisure-Time Physical Activity Questionnaire^90^. While the original questionnaire has been validated^91^, the modified version to be used in the present study has not been formally validated. However, the feasibility of the modified version has been confirmed at the large-scale 4-year lifestyle intervention study (DR's EXTRA)^92^. All ergo spirometry tests and HIIT sessions were conducted at Kuopio Research Institute of Exercise Medicine.

### Anthropometrics

Bodyweight was measured using digital scales. Waist circumference was measured at the midpoint between the lateral iliac and lowest rib to the nearest 0.5 cm during an exhale using a non-stretchable measuring tape. Bioelectrical impedance determined body composition (Inbody 720 body composition analyzer, USA) in a standing position after a 12-h fast. Each subject had to drink 200 mL water both in the evening and in the morning before the measurement. Blood pressure was measured twice by an automatic blood pressure monitor (Omron M 6 AC, Netherlands). The subjects had a 15 min rest before the measurement, and there was a 5 min break between the measurements. The mean of the measurements was used for data analysis.

### Resting energy expenditure

Indirect calorimetry was performed with a computerized flow-through canopy gas-analyzer system (Cosmed Quark, RMR, Italy) in the fasting state at the beginning and the end of the study (0 and 12 weeks). Energy expenditure and the rates of substrate oxidation were calculated according to Ferrannini^93^.

### Glucose monitoring

Glucose monitoring (Freestyle Libre, USA) was performed 2 weeks before the exercise started and at weeks 11 + 12. The subjects were instructed to perform 8–10 scans per day. After the 2-week monitoring period, the results were processed by a specific software from Freestyle Libre in order to estimate the daily fluctuation of blood glucose concentration.

### Magnetic resonance imaging

Liver fat was measured by nuclear MRI at the radiologic department at Kuopio University Hospital. Siemens Avanto_fit, NUMARIS/4; Syngo MR E11 and Siemens Aera, NUMARIS/4 machines were used to perform 2D axial fl2d6 sequence imaging to cover the whole liver. Images were pre-analyzed for clinical aspects at the Kuopio university hospital, and the final analysis was performed in Amsterdam Medical Centre via their special algorithm.

### Blood samples

Blood samples for clinical parameters were drawn at fasting state and were analyzed at Itä-Suomen laboratoriokeskuksen liikelaitoskuntayhtymä (ISLAB, Kuopio University Hospital). Insulin, ApoA1, and ApoB were analyzed at the University of Eastern Finland (UEF).

### Stool samples

Stool samples were collected in a plastic container with a lid by the subject him/herself while wearing gloves at weeks 0, 6, and 12. The sealed container was placed in an icebox filled with ice bags and brought to the research unit the next day. For weeks 6 and 12, an anaerobic generator paper bag was added. At the research unit, stool samples were directly homogenized, aliquoted, and frozen at − 80 °C without any detergents for further analysis. For the analysis, baseline and 12-week stool samples were analyzed.

### Urine samples

Urine was collected for 24 h in plastic containers at the beginning and end of the study to determine metabolite content. The samples were collected by the subject him/herself, brought to the research unit, and frozen at − 80 °C until the analysis.

### Adipose tissue biopsy

After an overnight fast and 30-min resting, adipose tissue (AT) samples were taken by open biopsy from subcutaneous abdominal AT before and after the intervention under sterile and local anesthesia (lidocaine 10 mg/mL). AT samples were washed twice with phosphate-buffered saline and immediately flash-frozen in liquid nitrogen. Samples were stored at − 80 °C for analysis.

### Statistics for clinical parameters

The normality of the clinical data was determined using a Q–Q plot. Continuous variables are expressed as mean and standard deviation (SD) for parametric measures. ALT, AST, insulin, TG, and γ-GT were log-transformed. Differences between the group were analyzed with a linear regression model, using age, gender, T2D status, and BMI as covariates. All descriptive statistical analyses were performed using SPSS statistical software (versions 25 and 27, IBM Corp, Armonk, NY). It was estimated that there would be a 2.38% decrease in the intrahepatic lipid content (IHL), as measured by MRS, in the HIIT intervention group compared to the control group. For this effect size, a significance level of 0.05 and a power of 80% was set.

### Non-targeted metabolite profiling analysis

#### Sample preparation

A total of four sample types were analyzed in this study—plasma, AT, urine, and stool. All samples were recoded and randomized using Wranglr (https://github.com/antonvsdata/wranglr), and the samples were prepared according to Klåvus et al.^94^. Briefly, plasma samples were prepared by adding 400 µL of cold acetonitrile to 100 µL of plasma, and urine samples were prepared by adding 200 µL of cold acetonitrile to 100 µL of the urine sample. The precipitated samples were filtered (Captiva ND filter plate 0.2 µm) by centrifuging for 5 min at 700×*g* at 4 °C. The filtered samples were kept at 4 °C until analysis. The AT and stool samples were homogenized by adding 80% v/v aqueous HPLC grade methanol in a ratio of 500 µL per 100 mg of sample for the metabolite extraction and protein precipitation using Bead Ruptor 24 Elite homogenizer at the speed 6 m/s at 0 ± 2 °C for 30 s. The samples were subsequently vortexed and centrifuged for 10 min at 4 °C and 20,000×*g*. The supernatant was collected and filtered (Captiva ND filter plate 0.2 µm) by centrifuging for 5 min at 700×*g* at 4 °C into HPLC vials and kept at 4 °C until analysis. Aliquots of 2 µL were taken from all sample types separately, mixed in one tube (per sample type), and used as the quality control samples (for that sample type) in the analysis.

#### LC–MS analysis

The non-targeted metabolic profiling analysis was performed as described by Klåvus et al.^94^. Briefly, ultra-high performance liquid chromatography (Vanquish Flex UHPLC system, Thermo Scientific, Bremen, Germany) coupled to high-resolution mass spectrometry (Q Exactive Focus, Thermo Scientific, Bremen, Germany) was used. Here, each sample (1 µL per injection) was analyzed using reversed-phase (RP) chromatography (Zorbax Eclipse XDBC18, 2.1 × 100 mm, 1.8 μm, Agilent Technologies, Palo Alto, CA, USA) maintained at 40 °C. The mobile phase consisted of water (solution A) and methanol (solution B), both containing 0.1% v/v formic acid. The elution gradient profile was as follows (t [min], %B): (0, 2), (10, 100), (14.5, 100), (14.51, 2), (16.5, 2). The data were acquired in both positive (ESI+) and negative (ESI−) electrospray ionization modes. Quality control samples were injected at the beginning of the analysis and after every 12 samples. All data were acquired in centroid mode using FreeStyle 1.3 (Thermo Fisher Scientific).

Additionally, 1290 Infinity Binary UPLC coupled with a 6540 UHD Accurate-Mass QTOF (Agilent Technologies, Santa Clara, CA, USA) was used for LC–MS with HILIC chromatography. Acquity UPLC BEH amide column (2.1 × 100 mm, 1.7 μm; Waters Corporation) with an injection volume of 3 μL was used for the HILIC separation. The mobile phases were 50% acetonitrile (vol:vol; eluent A) and 90% acetonitrile (vol:vol; eluent B), respectively, both containing 20 mmol/L ammonium formate, pH 3 (Sigma-Aldrich). The gradient was: 0–2.5 min, 100% B; 2.5–10 min, 100% B → 0% B; 10–10.1 min, 0% B → 100% B; 10.1–14 min, 100% B, and flow rate 0.6 mL/min. After each chromatographic separation, the ionization was carried out using jet stream electrospray ionization (ESI) in the positive and negative mode, yielding four data files per sample. The collision energies for the MS/MS analysis were chosen as 10, 20, and 40 V for compatibility with the spectral databases. Quality control samples were injected at the beginning of the analysis and after every 12 samples. The data acquisition software was MassHunter Acquisition B.07.00 (Agilent Technologies). Additionally, 1290 Infinity Binary UPLC coupled with a 6540 UHD Accurate-Mass QTOF (Agilent Technologies, Santa Clara, CA, USA) was used for LC–MS with HILIC chromatography. Acquity UPLC BEH amide column (2.1 × 100 mm, 1.7 μm; Waters Corporation) with an injection volume of 3 μL was used for the HILIC separation. The mobile phases were 50% acetonitrile (vol:vol; eluent A) and 90% acetonitrile (vol:vol; eluent B), respectively, both containing 20 mmol/L ammonium formate, pH 3 (Sigma-Aldrich). The gradient was: 0–2.5 min, 100% B; 2.5–10 min, 100% B → 0% B; 10–10.1 min, 0% B → 100% B; 10.1–14 min, 100% B, and flow rate 0.6 mL/min. After each chromatographic separation, the ionization was carried out using jet stream electrospray ionization (ESI) in the positive and negative mode, yielding four data files per sample. The collision energies for the MS/MS analysis were chosen as 10, 20, and 40 V for compatibility with the spectral databases^95–97^. Quality control samples were injected at the beginning of the analysis and after every 12 samples. The data acquisition software was MassHunter Acquisition B.07.00 (Agilent Technologies).

#### Peak picking and alignment

After the conversion of the raw instrumental data (*.d files) to ABF format using Reifycs Abf Converter (https://www.reifycs.com/AbfConverter), MS-DIAL (Version 4.24)^98^ was employed for automated peak picking and alignment. The parameters were set according to Klåvus et al.^94^. After peak picking, the alignment result across all sample types as peak areas was exported into Microsoft Excel and henceforth underwent data pre-processing. A total of 134,313 features were obtained from the peak-picking from the four analytical modes.

#### Data pre-processing

Data pre-processing was done separately for each sample matrices and analytical modes using R version 3.6.1^99^. Briefly, signals present in less than 80% of the samples in all groups and with a detection rate less than 70% of the pooled QC samples were excluded. Thereafter, they were corrected for intensity drift^94^. After drift correction, QC samples were removed, and low-quality signals were flagged according to the guidelines in Klåvus et al.^94^. After that, the missing values in the high-quality signals were imputed using random forest imputation. Missing values in low-quality signals were imputed with zeroes since many of the low-quality signals showed a high proportion of missingness, which would impede random forest imputation.

#### Statistical analysis

All statistical analyses for metabolite signals were performed using R version 3.6.1^99^. Briefly, a feature-wise linear mixed model was fit to spot the differential metabolites using R packages lme4^100^ and lmer Test^101^. Feature levels were used as the dependent variables. The effects of the intervention, time, and their interaction were modeled as fixed effects, and subject ID was used as a random effect. The interaction term reflects the difference in changes during the intervention between the control and the exercise intervention group. The significance of the regression coefficients was tested using a *t* test with Satterthwaite's approximation for degrees of freedom. Confidence intervals were constructed using parametric bootstrapping percentile intervals with 1000 simulations. A raw p-value < 0.05 was used as a criterion for further investigation and annotation of signals. Further, we also selected only those features which had an MS/MS spectrum available and had an average peak area of at least 10,000 per sample type, including altogether 5067 features (469 plasma, 670 AT, 3020 urine, and 1008 stool).

#### Metabolite identification

The signals chosen for further investigation were annotated using MS-DIAL Version 4.24^98^ by comparing the exact m/z, retention time, and MS/MS fragmentation patterns against our in-house standard library. Further, additional searches in online MS spectral databases were also performed^95,96,102,103^. Additionally, MS-FINDER Version 3.50 was used to characterize the unknowns^104^. Moreover, the vendor software—Agilent MassHunter Qualitative Analysis B.07.00 and FreeStyle 1.3 were used for the exploration of raw data extracted ion chromatograms (EICs) and MS/MS fragmentation spectra.

Following annotation, multivariate analysis was performed in R Version 3.6.2^99^. A heatmap representing Spearman's correlations was plotted using ClustVis^105^ individually for delta change of the variables from baseline to post-intervention for each of the sample matrices and volcano plots of standardized effect size and p-values from the linear mixed models were created using VolcaNoseR^106^. The top 25 most significantly different metabolites for each sample matrix were then further analyzed and discussed. For pathway analyses, MetaboAnalyst version 5.0 was used with *homo Sapiens* KEGG as the reference library and all compounds in the selected pathways as the reference metabolome^18^. The over-representation analysis was performed using Fisher's exact test and the pathway topology using relative-betweenness centrality.

### Study approval

The study was approved by the Research Ethics Committee of the Northern Savo Hospital District as of April 1st, 2019 (approval #: 565/2019), and written informed consent was obtained from all participants. The study including all methods was performed in accordance with the declaration of Helsinki and the guidelines and regulations of the review board. The trial is registered in ClinicalTrials.gov (NCT03995056, 10.06.2019).

## Supplementary Information

Supplementary Information 1.

Supplementary Information 2.

## References

[1] Maurice J, Manousou P (2018). Non-alcoholic fatty liver disease. Clin. Med..

[2] Younossi ZM (2016). Global epidemiology of nonalcoholic fatty liver disease—Meta-analytic assessment of prevalence, incidence, and outcomes. Hepatology.

[3] Farrell GC, Larter CZ (2006). Nonalcoholic fatty liver disease: From steatosis to cirrhosis. Hepatology.

[4] Byrne CD, Targher G (2015). NAFLD: A multisystem disease. J. Hepatol..

[5] Zhang H-J (2016). Effects of moderate and vigorous exercise on nonalcoholic fatty liver disease: A randomized clinical trial. JAMA Intern. Med..

[6] Golabi P (2016). Effectiveness of exercise in hepatic fat mobilization in nonalcoholic fatty liver disease: Systematic review. World J. Gastroenterol..

[7] Van Der Heijden GJ (2010). A 12-week aerobic exercise program reduces hepatic fat accumulation and insulin resistance in obese, hispanic adolescents. Obesity.

[8] Keating SE, Hackett DA, George J, Johnson NA (2012). Exercise and non-alcoholic fatty liver disease: A systematic review and meta-analysis. J. Hepatol..

[9] Davoodi, M., Moosavi, H. & Nikbakht, M. The effect of eight weeks selected aerobic exercise on liver parenchyma and liver enzymes (AST, ALT) of fat liver patients. *J. Shahrekord Univ. Med. Sci.* **14** (2012).

[10] Babu AF (2021). Positive effects of exercise intervention without weight loss and dietary changes in NAFLD-related clinical parameters: A systematic review and meta-analysis. Nutrients.

[11] Daskalaki E (2015). A study of the effects of exercise on the urinary metabolome using normalisation to individual metabolic output. Metabolites.

[12] Zhao X (2018). Response of gut microbiota to metabolite changes induced by endurance exercise. Front. Microbiol..

[13] Kalhan SC (2011). Plasma metabolomic profile in nonalcoholic fatty liver disease. Metabolism.

[14] Gorden DL (2015). Biomarkers of NAFLD progression: A lipidomics approach to an epidemic 1. J. Lipid Res..

[15] de Mello VD (2021). Serum aromatic and branched-chain amino acids associated with NASH demonstrate divergent associations with serum lipids. Liver Int..

[16] Dong S (2017). Urinary metabolomics analysis identifies key biomarkers of different stages of nonalcoholic fatty liver disease. World J. Gastroenterol..

[17] Chu H, Duan Y, Yang L, Schnabl B (2019). Small metabolites, possible big changes: A microbiota-centered view of non-alcoholic fatty liver disease. Gut.

[18] Chong J, Yamamoto M, Xia J (2019). MetaboAnalystR 2.0: From raw spectra to biological insights. Metabolites.

[19] Marchesini G (2016). EASL-EASD-EASO clinical practice guidelines for the management of non-alcoholic fatty liver disease. J. Hepatol..

[20] Cheng S (2017). Effect of aerobic exercise and diet on liver fat in pre-diabetic patients with non-alcoholic-fatty-liver-disease: A randomized controlled trial. Sci. Rep..

[21] Pugh CJA (2014). Exercise training reverses endothelial dysfunction in nonalcoholic fatty liver disease. Am. J. Physiol. Heart Circ. Physiol..

[22] Cuthbertson DJ (2016). Dissociation between exercise-induced reduction in liver fat and changes in hepatic and peripheral glucose homoeostasis in obese patients with non-alcoholic fatty liver disease. Clin. Sci. (Lond).

[23] Church TS, LaMonte MJ, Barlow CE, Blair SN (2005). Cardiorespiratory fitness and body mass index as predictors of cardiovascular disease mortality among men with diabetes. Arch. Intern. Med..

[24] Wei M (1999). The association between cardiorespiratory fitness and impaired fasting glucose and type 2 diabetes mellitus in men. Ann. Intern. Med..

[25] Evans PL, McMillin SL, Weyrauch LA, Witczak CA (2019). Regulation of skeletal muscle glucose transport and glucose metabolism by exercise training. Nutrients.

[26] Ross R (2020). Waist circumference as a vital sign in clinical practice: A Consensus Statement from the IAS and ICCR Working Group on Visceral Obesity. Nat. Rev. Endocrinol..

[27] Zadeh-Vakili A, Tehrani FR, Hosseinpanah F (2011). Waist circumference and insulin resistance: A community based cross sectional study on reproductive aged Iranian women. Diabetol. Metab. Syndr..

[28] Kashiwagi R (2017). Effective waist circumference reduction rate necessary to avoid the development of type 2 diabetes in Japanese men with abdominal obesity. Endocr. J..

[29] Johnson NA (2009). Aerobic exercise training reduces hepatic and visceral lipids in obese individuals without weight loss. Hepatology.

[30] Winn NC (2018). Energy-matched moderate and high intensity exercise training improves nonalcoholic fatty liver disease risk independent of changes in body mass or abdominal adiposity—A randomized trial. Metabolism.

[31] Kaartinen N (2020). The Finnish National Dietary Survey in Adults and Elderly (FinDiet 2017). EFSA Support. Publ..

[32] Shou J, Chen PJ, Xiao WH (2019). The effects of BCAAs on insulin resistance in athletes. J. Nutr. Sci. Vitaminol. (Tokyo).

[33] Herman MA, She P, Peroni OD, Lynch CJ, Kahn BB (2010). Adipose tissue branched chain amino acid (BCAA) metabolism modulates circulating BCAA levels. J. Biol. Chem..

[34] Lynch CJ (2002). Leucine is a direct-acting nutrient signal that regulates protein synthesis in adipose tissue. Am. J. Physiol. Endocrinol. Metab..

[35] Zhang L (2020). Leucine supplementation: A novel strategy for modulating lipid metabolism and energy homeostasis. Nutrients.

[36] Cai H, Dong L, Liu F (2016). Recent advances in adipose mTOR signaling and function: Therapeutic prospects. Trends Pharmacol. Sci..

[37] Bruckbauer A, Zemel MB (2011). Effects of dairy consumption on SIRT1 and mitochondrial biogenesis in adipocytes and muscle cells. Nutr. Metab..

[38] Duan Y (2015). Nutritional and regulatory roles of leucine in muscle growth and fat reduction. Front. Biosci. (Landmark Ed.).

[39] Sun X, Zemel MB (2009). Leucine modulation of mitochondrial mass and oxygen consumption in skeletal muscle cells and adipocytes. Nutr. Metab..

[40] Bartelt A, Heeren J (2014). Adipose tissue browning and metabolic health. Nat. Rev. Endocrinol..

[41] Ma Q (2020). Threonine, but not lysine and methionine, reduces fat accumulation by regulating lipid metabolism in obese mice. J. Agric. Food Chem..

[42] José V (2019). The role of PGC-1 α/UCP2 signaling in the beneficial effects of physical exercise on the brain. Front. Neurosci..

[43] Lin J, Handschin C, Spiegelman BM (2005). Metabolic control through the PGC-1 family of transcription coactivators. Cell Metab..

[44] Li J (2020). Muscle metabolomics analysis reveals potential biomarkers of exercise - dependent improvement of the diaphragm function in chronic obstructive pulmonary disease. Int. J. Mol. Med..

[45] Zouhal H, Jacob C, Delamarche P, Gratas-Delamarche A (2008). Catecholamines and the effects of exercise, training and gender. Sports Med..

[46] Vargovic P, Ukropec J, Laukova M, Cleary S, Manz B, Pacak K, Kvetnansky R (2011). Adipocytes as a new source of catecholamine production. FEBS Lett..

[47] Bazzano M (2020). Exercise induced changes in salivary and serum metabolome in trained standardbred, assessed by1H-NMR. Metabolites.

[48] Tabone M (2021). The effect of acute moderate-intensity exercise on the serum and fecal metabolomes and the gut microbiota of cross-country endurance athletes. Sci. Rep..

[49] Amaretti A (2019). Profiling of protein degraders in cultures of human gut microbiota. Front. Microbiol..

[50] Chambers ES, Preston T, Frost G, Morrison DJ (2018). Role of gut microbiota-generated short-chain fatty acids in metabolic and cardiovascular health. Curr. Nutr. Rep..

[51] Heimann E, Nyman M, Pålbrink A-K, Lindkvist-Petersson K, Degerman E (2016). Branched short-chain fatty acids modulate glucose and lipid metabolism in primary adipocytes. Adipocyte.

[52] Meadows JA, Wargo MJ (2015). Carnitine in bacterial physiology and metabolism. Microbiology (United Kingdom).

[53] Mingorance C, Gonzalez Del Pozo M, Dolores Herrera M, Alvarez De Sotomayor M (2009). Oral supplementation of propionyl-l-carnitine reduces body weight and hyperinsulinaemia in obese Zucker rats. Br. J. Nutr..

[54] Mingorance C, Rodriguez-Rodriguez R, Justo ML, Herrera MD, de Sotomayor MA (2011). Pharmacological effects and clinical applications of propionyl-l-carnitine. Nutr. Rev..

[55] Perichon, R., Bell, Lauren, N., Wulff, J., Nguyen, U. T. & Watkins, S. Biomarkers for fatty liver disease and methods using the same (2016).

[56] Mardinoglu A (2017). Personal model-assisted identification of NAD+ and glutathione metabolism as intervention target in NAFLD. Mol. Syst. Biol..

[57] Lustgarten MS, Price LL, Chalé A, Fielding RA (2014). Metabolites related to gut bacterial metabolism, peroxisome proliferator-activated receptor-alpha activation, and insulin sensitivity are associated with physical function in functionally-limited older adults. Aging Cell.

[58] Hendrikx T, Schnabl B (2019). Indoles: metabolites produced by intestinal bacteria capable of controlling liver disease manifestation. J. Intern. Med..

[59] Lin Y-H (2019). Aryl hydrocarbon receptor agonist indigo protects against obesity-related insulin resistance through modulation of intestinal and metabolic tissue immunity. Int. J. Obes..

[60] Manaf FA (2018). Characterizing the plasma metabolome during and following a maximal exercise cycling test. J. Appl. Physiol..

[61] Kartsoli S, Kostara CE, Tsimihodimos V, Bairaktari ET, Christodoulou DK (2020). Lipidomics in non-alcoholic fatty liver disease. World J. Hepatol..

[62] Summers SA (2006). Ceramides in insulin resistance and lipotoxicity. Prog. Lipid Res..

[63] Li J (2019). Serum metabolomic analysis of the effect of exercise on nonalcoholic fatty liver disease. Endocr. Connect..

[64] Bergman BC (2015). Serum sphingolipids: Relationships to insulin sensitivity and changes with exercise in humans. Am. J. Physiol. Endocrinol. Metab..

[65] Montgomery MK (2016). Regulation of glucose homeostasis and insulin action by ceramide acyl-chain length: A beneficial role for very long-chain sphingolipid species. Biochim. Biophys. Acta Mol. Cell Biol. Lipids.

[66] Zhou Y (2016). Noninvasive detection of nonalcoholic steatohepatitis using clinical markers and circulating levels of lipids and metabolites. Clin. Gastroenterol. Hepatol. Off. Clin. Pract. J. Am. Gastroenterol. Assoc..

[67] Draijer LG (2020). Lipidomics in nonalcoholic fatty liver disease: Exploring serum lipids as biomarkers for pediatric nonalcoholic fatty liver disease. J. Pediatr. Gastroenterol. Nutr..

[68] Kasumov T (2015). Improved insulin sensitivity after exercise training is linked to reduced plasma C14:0 ceramide in obesity and type 2 diabetes. Obesity.

[69] Hoene M (2016). Muscle and liver-specific alterations in lipid and acylcarnitine metabolism after a single bout of exercise in mice. Sci. Rep..

[70] Gottlieb A, Canbay A (2019). Why bile acids are so important in non-alcoholic fatty liver disease (NAFLD) progression. Cells.

[71] Mouzaki M (2016). Bile acids and dysbiosis in non-alcoholic fatty liver disease. PLoS One.

[72] Ferslew BC (2015). Altered bile acid metabolome in patients with nonalcoholic steatohepatitis. Dig. Dis. Sci..

[73] Aranha MM (2008). Bile acid levels are increased in the liver of patients with steatohepatitis. Eur. J. Gastroenterol. Hepatol..

[74] Targher G, Corey KE, Byrne CD, Roden M (2021). The complex link between NAFLD and type 2 diabetes mellitus—Mechanisms and treatments. Nat. Rev. Gastroenterol. Hepatol..

[75] Schranner D, Kastenmüller G, Schönfelder M, Römisch-Margl W, Wackerhage H (2020). Metabolite concentration changes in humans after a bout of exercise: A systematic review of exercise metabolomics studies. Sports Med. Open.

[76] Morville T (2018). Divergent effects of resistance and endurance exercise on plasma bile acids, FGF19, and FGF21 in humans. JCI Insight.

[77] Danese E (2017). Analytical evaluation of three enzymatic assays for measuring total bile acids in plasma using a fully-automated clinical chemistry platform. PLoS One.

[78] Wertheim BC (2009). Physical activity as a determinant of fecal bile acid levels. Cancer Epidemiol. Biomark. Prev..

[79] Kudchodkar BJ, Sodhi HS, Mason DT, Borhani NO (1977). Effects of acute caloric restriction on cholesterol metabolism in man. Am. J. Clin. Nutr..

[80] Morville T, Sahl RE, Moritz T, Helge JW, Clemmensen C (2020). Plasma metabolome profiling of resistance exercise and endurance exercise in humans. Cell Rep..

[81] Alzharani MA, Alshuwaier GO, Aljaloud KS, Al-Tannak NF, Watson DG (2020). Metabolomics profiling of plasma, urine and saliva after short term training in young professional football players in Saudi Arabia. Sci. Rep..

[82] Kim YS, Li XF, Kang KH, Ryu B, Kim SK (2014). Stigmasterol isolated from marine microalgae *Navicula incerta* induces apoptosis in human hepatoma HepG2 cells. BMB Rep..

[83] Casal JJ, Bollini M, Lombardo ME, Bruno AM (2016). Thalidomide analogues: Tumor necrosis factor-alpha inhibitors and their evaluation as anti-inflammatory agents. Eur. J. Pharm. Sci..

[84] Gabay O (2010). Stigmasterol: a phytosterol with potential anti-osteoarthritic properties. Osteoarthr. Cartil..

[85] Li C, Liu Y, Xie Z, Lu Q, Luo S (2015). Stigmasterol protects against Ang II-induced proliferation of the A7r5 aortic smooth muscle cell-line. Food Funct..

[86] Saleem M (2020). Association between sphingolipids and cardiopulmonary fitness in coronary artery disease patients undertaking cardiac rehabilitation. J. Gerontol. Ser. A.

[87] Hallsworth K (2015). Modified high-intensity interval training reduces liver fat and improves cardiac function in non-alcoholic fatty liver disease: A randomized controlled trial. Clin. Sci. (Lond).

[88] Tornvall G (1963). Assessment of Physical Capabilities.

[89] Physical activity guidelines for Americans, Vol. 53 25. https://health.gov/our-work/physical-activity/current-guidelines (2018). 19090067

[90] Taylor HL (1978). A questionnaire for the assessment of leisure time physical activities. J. Chron. Dis..

[91] Bonnefoy M (2001). Simultaneous validation of ten physical activity questionnaires in older men: A doubly labeled water study. J. Am. Geriatr. Soc..

[92] Hakola L (2015). Moderators of maintained increase in aerobic exercise among aging men and women in a 4-year randomized controlled trial: The DR’s EXTRA study. J. Phys. Act. Health.

[93] Ferrannini E (1988). The theoretical bases of indirect calorimetry: A review. Metabolism.

[94] Klåvus A (2020). “Notame”: Workflow for non-targeted LC–MS metabolic profiling. Metabolites.

[95] Smith CA (2005). METLIN: A metabolite mass spectral database. Ther. Drug Monit..

[96] Wishart DS (2007). HMDB: The Human Metabolome Database. Nucleic Acids Res..

[97] Kim S (2016). PubChem substance and compound databases. Nucleic Acids Res..

[98] Hiroshi T (2015). MS-DIAL: Data-independent MS/MS deconvolution for comprehensive metabolome analysis. Nat. Methods.

[99] The R Core Team. R: The R Project for Statistical Computing. https://www.r-project.org/ (2019).

[100] Bates, D., Mächler, M., Bolker, B. & Walker, S. Fitting linear mixed-effects models using lme4. *J. Stat. Softw.* **1**(1) (2015).

[101] Kuznetsova, A., Brockhoff, P. B. & Christensen, R. H. B. lmerTest Package: Tests in linear mixed effects models. *J. Stat. Software ***1**(13) (2017).

[102] The LIPID MAPS^®^ Lipidomics Gateway.

[103] MetaboAtlas21. https://metaboatlas21.metabolomics.fgu.cas.cz/

[104] Tsugawa H (2016). Hydrogen rearrangement rules: Computational MS/MS fragmentation and structure elucidation using MS-FINDER software. Anal. Chem..

[105] Metsalu T, Vilo J (2015). ClustVis: A web tool for visualizing clustering of multivariate data using Principal Component Analysis and heatmap. Nucleic Acids Res..

[106] Goedhart J, Luijsterburg MS (2020). VolcaNoseR is a web app for creating, exploring, labeling and sharing volcano plots. Sci. Rep..

