# Supplementary material for: Effects of exercise on NAFLD using non-targeted metabolomics in adipose tissue, plasma, urine, and stool
Source: Sci Rep. 2022 Apr 20;12:6485. doi: 10.1038/s41598-022-10481-9 (PMC9019539; doi:10.1038/s41598-022-10481-9)
Supplement: Supplementary file 2 — Supplementary Information 2. [file 41598_2022_10481_MOESM2_ESM.docx]

**Supplements:**

***Supplementary Table 1.*** *Food parameters*

| *Characteristics* | *control group n=22* | | *intervention group n=20* | | *Difference between the groups* |
| --- | --- | --- | --- | --- | --- |
|  | **Week 0** | **Week 12** | **Week 0** | **Week 12** | **p-value^1^** |
| *Energy, KJ* | 9509 ± 2230 | 8817 ± 2222 | 8297 ± 1793 | 8296 ± 1902 | 0.216 |
| *Energy, kcal* | 2272 ± 533 | 2106 ± 531 | 1982 ± 428 | 1981 ± 455 | 0.216 |
| *Carbohydrates, % ** | 41.8 ± 4.5 | 41.8 ± 5.1 | 40.3 ± 5.3 | 41.8 ± 5.6 | 0.256 |
| *Protein, % ** | 17.1 ± 2.6 | 17.0 ± 3.2 | 18.5 ± 3.3 | 18.0 ± 3.6 | 0.518 |
| *Fat, % ** | 37.3 ± 5.0 | 37.3 ± 5.0 | 37.1 ± 6.7 | 36.5 ± 5.0 | 0.776 |
| *Saturated fat, %** | 13.3 ± 3.0 | 13.3 ± 2.4 | 13.8 ± 2.9 | 13.1 ± 3.1 | 0.516 |
| *Mono-unsaturated fat, %** | 13.6 ± 2.4 | 13.0 ± 2.4 | 13.5 ± 2.7 | 13.0 ± 2.2 | 0.931 |
| *Poly-unsaturated fat, %** | 6.5 ± 1.7 | 6.8 ± 2.0 | 6.2 ± 1.3 | 6.5 ± 1.5 | 0.917 |
| *Cholesterol, mg* | 297 ± 103 | 249 ± 109 | 288 ± 94 | 265 ± 83 | 0.589 |
| *Fibre, g* | 26.4 ± 9.7 | 24.8 ± 7.6 | 24.0 ± 7.4 | 24.7 ± 7.5 | 0.11 |
| *Alcohol,* %* | 1.0 ± 2.1 | 1.4 ± 2.7 | 1.3 ± 3.6 | 0.7 ± 2.3 | 0.303 |

*4-d food record at baseline, 4-d food record at the last week of intervention, * of energy intake (kcal),* ***^1^*** *Linear regression model (Covariates: T2D, gender, age, BMI); Values are means ± SD*

***Supplementary Table 2.*** *Body composition and bioimpedance results*

| *Characteristics* | *control group n=22* | | *intervention group n=20* | | *Difference between the groups* |
| --- | --- | --- | --- | --- | --- |
|  | **Week 0** | **Week 12** | **Week 0** | **Week 12** | **p-value^1^** |
| *Intracellular Water, kg* | 25.9 ± 5.2 | 25.7 ± 5.2 | 24.6 ± 4.7 | 24.3 ± 4.8 | 0.418 |
| *Extracellular Water, kg* | 15.71 ± 3.1 | 15.7 ± 3.0 | 15.0 ± 2.8 | 14.9 ± 2.9 | 0.299 |
| *Protein Mass, kg* | 11.2 ± 2.2 | 11.1 ± 2.3 | 10.6 ± 2.1 | 10.5 ± 2.1 | 0.526 |
| *Fat Mass, %* | 33.9 ± 9.0 | 34.5 ± 8.5 | 35.8 ± 5.1 | 36.3 ± 4.8 | 0.766 |
| *Fat Mass, kg* | 29.1 ± 10.2 | 30.1 ± 9.8 | 30.3 ± 7.8 | 30.6 ± 7.6 | 0.872 |
| *Muscle Mass, kg* | 31.8 ± 6.8 | 31.6 ± 6.7 | 30.1 ± 6.2 | 29.7 ± 6.2 | 0.403 |
| *Visceral Fat Area, cm^2^* | 145.1 ± 54.3 | 146.1 ± 52.4 | 148.7 ± 42.3 | 149.0 ± 42.0 | 0.893 |
| *RMR, kcal/day* | 1582 ± 270 | 1581 ± 266 | 1533 ± 288 | 1564.2 ± 292 | 0.377 |
| *VO****_2_****, ml/min resting state* | 229.7 ± 40.9 | 230.0 ± 39.1 | 223.0 ± 42.4 | 227.3 ± 41.7 | 0.535 |
| *VCO****_2_****, ml/min resting state* | 186.1 ± 29.2 | 185.1 ± 31.3 | 179.4 ± 32.8 | 183.6 ± 37.4 | 0.133 |

*RMR: resting metabolic rate;* ***^1^*** *Linear regression model (Covariates: T2D, gender, age, BMI); Values are means ± SD*

***Supplementary Table 3***

Significantly different metabolites across adipose tissue, plasma, urine, and stool with their observed LC-MS characteristics. Identification level 1 = identified with a reference standard; Identification level 2 = putatively annotated based on publicly available exact m/z and MS/MS spectra

*(Provided as an excel sheet)*

***Supplementary Table 4****. The exercise training program in the high-intensity interval group*

| Study months | **0–1** |  | **1–2** | **2-3** |
| --- | --- | --- | --- | --- |
| *High-intensity interval training* |  |  |  |  |
| Duration of warm-up, min | 5 |  | 5 | 5 |
| High-intensity interval training frequency/week | 2 |  | 2 | 2 |
| Intensity of work phase, % maxW_4_ | 85 |  | 85 | 85 |
| Intensity of recovery phase, % maxW_4_ | 20 |  | 20 | 20 |
| Interval sets, number | 5 |  | 5 | 5 |
| Duration of the interval, seconds | 120-160 |  | 160-200 | 200-240 |
| Recovery between sets, minutes | 3 |  | 3 | 3 |
| Intensity of warm-up, % maxW_4_ | 30 |  | 30 | 30 |
| Intensity of cool-down, % maxW_4_ | 20 |  | 20 | 20 |
| Duration of cool-down, min | 5 |  | 5 | 5 |
| *Additional home-based aerobic training* |  |  |  |  |
| Duration, min/week | 140 |  | 120 | 100 |

maxW4 refers to the hypothetical workload sustainable for 4 minutes


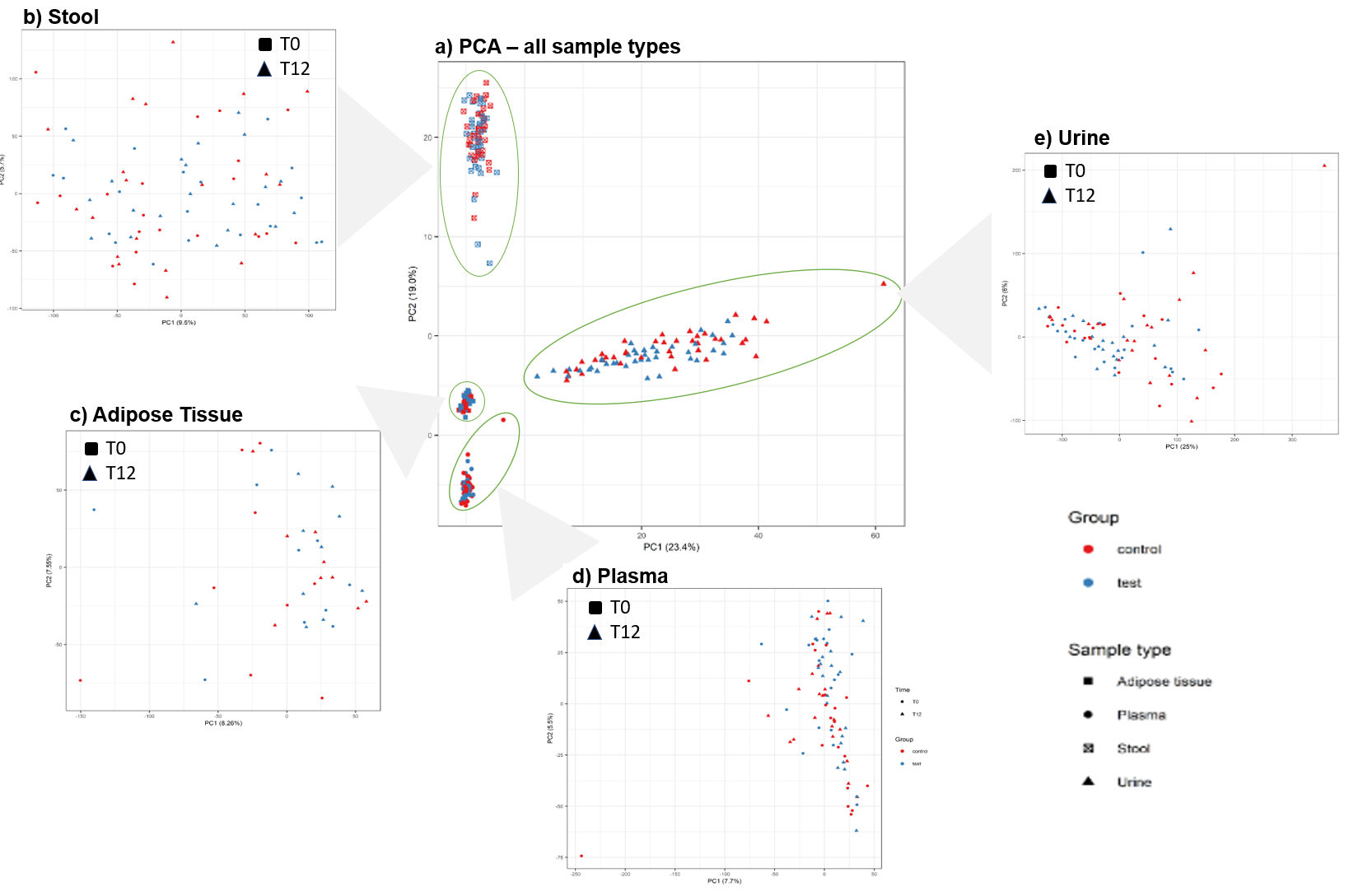


***Supplementary Figure 1****: Principal component analysis (PCA) of untargeted metabolomics data from four different sample matrices. a) PCA of all analysed samples.; b) Stool; c) Adipose tissue; d) Plasma; e) Urine*


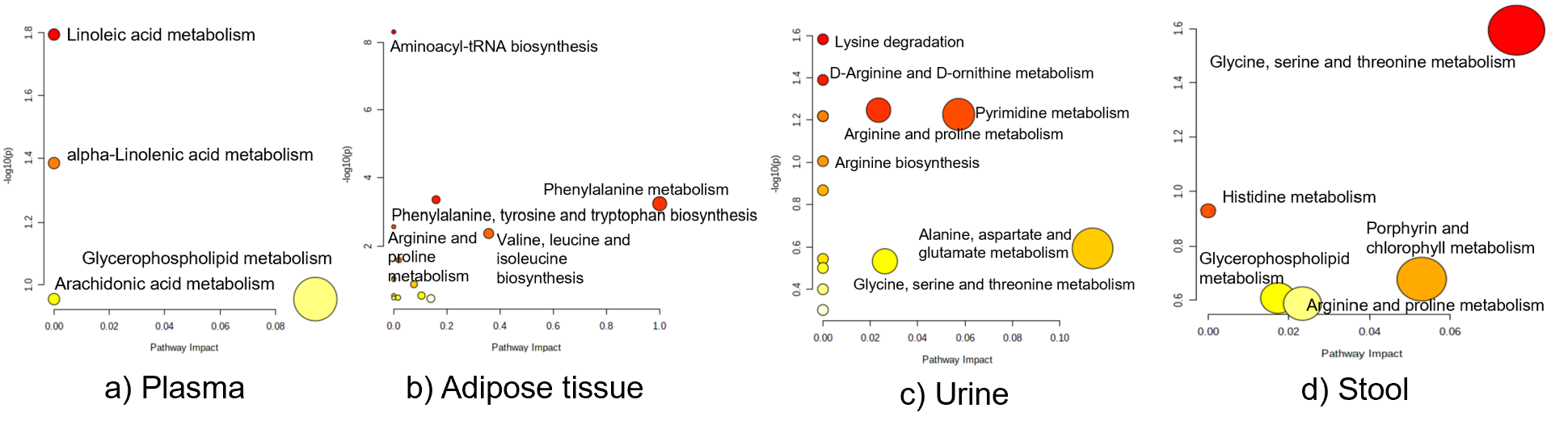


***Supplementary Figure 2:*** *Pathway analysis with MetaboAnalyst with p < 0.05. The Y-axis is the –log10 p values from the pathway enrichment analysis. The X-axis is the pathway impact values from pathway topology analysis. The node colors and radii are based on p values and pathway impact values, respectively. Color intensities increase with reducing p values, and the sizes of the circles increase with increased pathway impacts.*


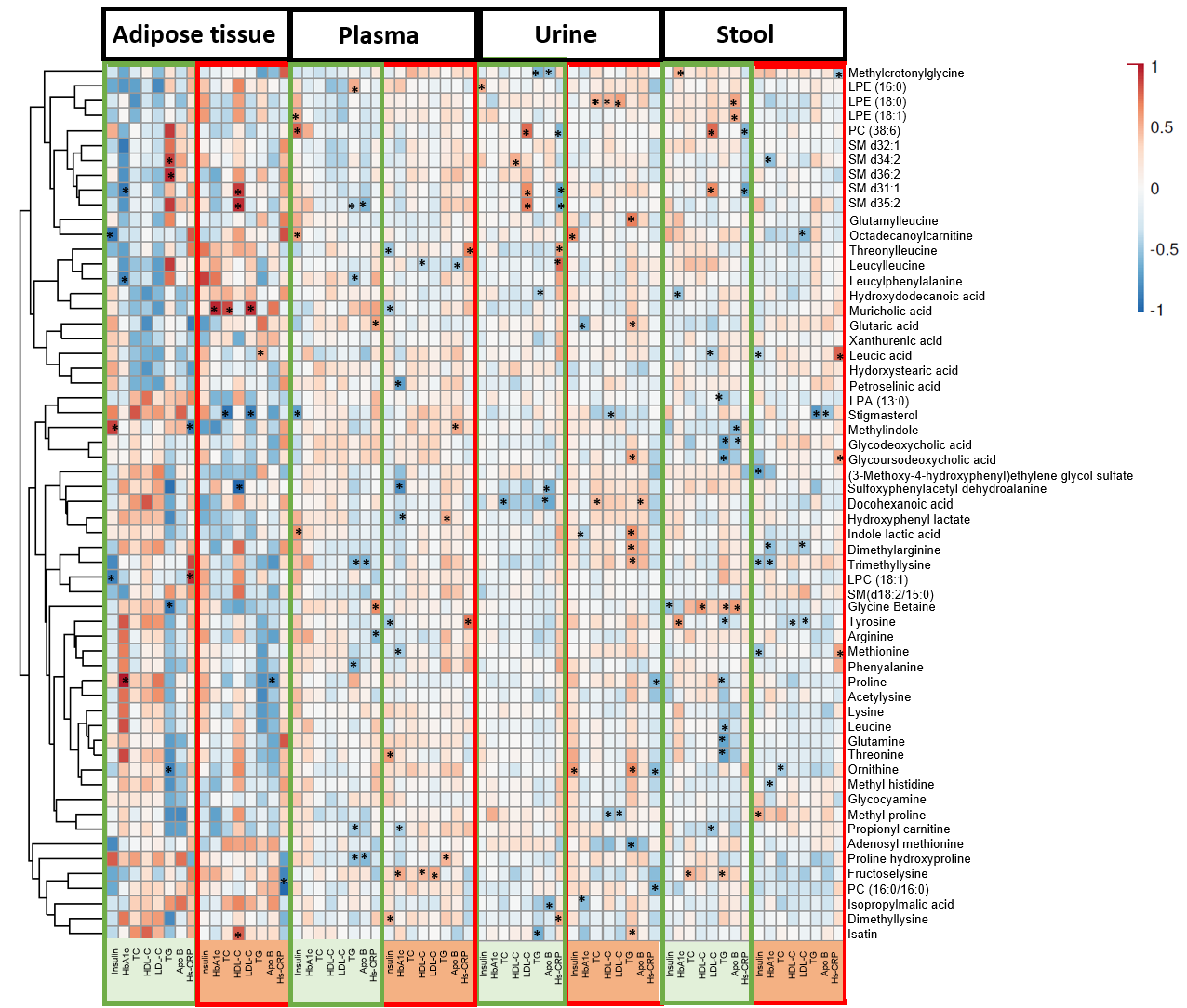


***Supplementary Figure 3:*** *Heatmap representing the Spearman's correlations (adjusted for age, gender, BMI, and T2D) between the glucose and lipid related clinical parameters (column-wise) and the top significantly different metabolites identified in all sample matrices combined (row-wise) using delta change from baseline to post intervention. The color of the cells indicates the strength of the relationship (*r_s_*). The cells marked with asterisks (*) demonstrate significant correlations (p<0.05). Green sidebars indicate the control group, and red sidebars represent the exercise intervention group. Abbreviations: HbA1C: glycated hemoglobin; TC: total cholesterol; HDL-C: high-density lipoprotein cholesterol; LDL-C: low-density lipoprotein cholesterol; TG: triglyceride; ApoB – Apolipoprotein B; Hs-CRP: high-sensitive C-reactive protein*


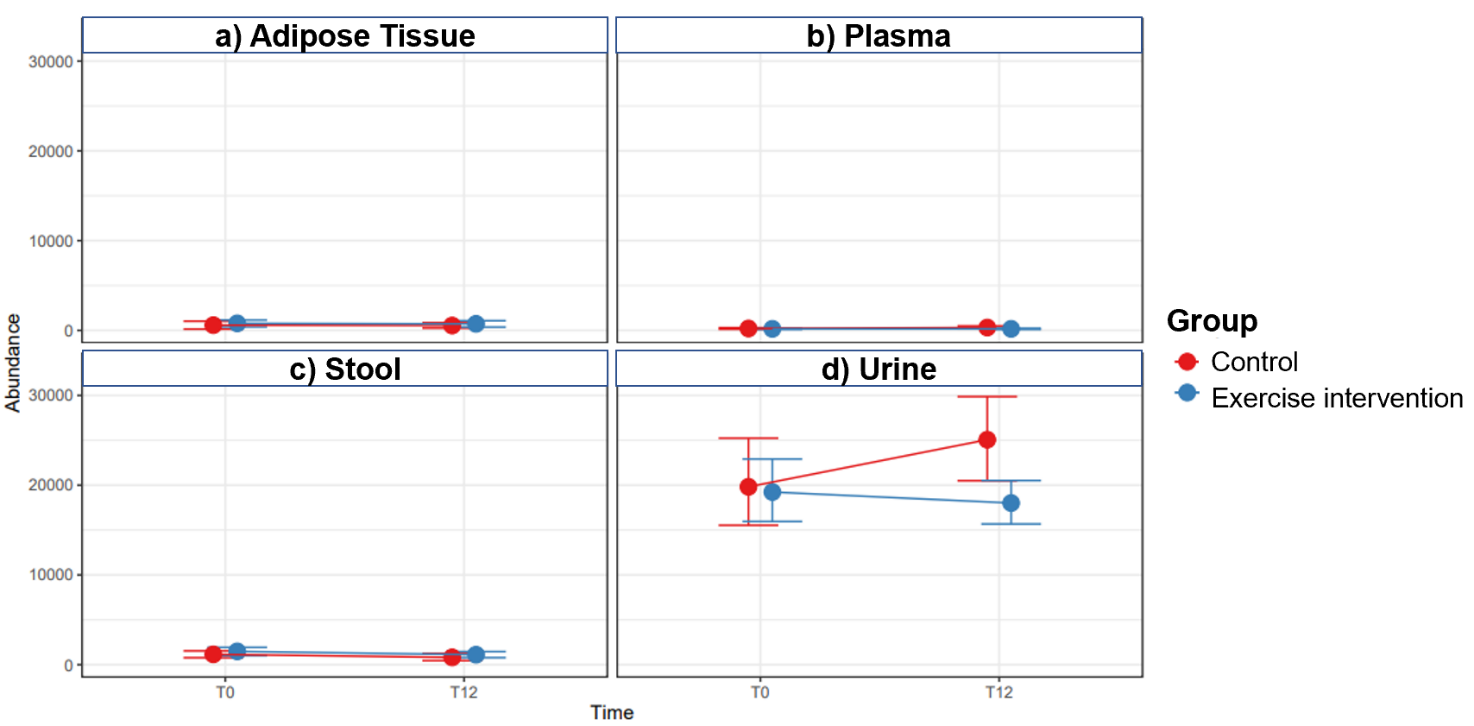


***Supplementary Figure 4:*** *Line plots depicting glycine levels across the four sample matrices in the control and exercise intervention groups between baseline and 12 weeks of intervention. a) Adipose tissue, b) Plasma, c) Stool, d) Urine*
